# CD25890, a conserved protein that modulates sporulation initiation in *Clostridioides difficile*

**DOI:** 10.1038/s41598-021-86878-9

**Published:** 2021-04-12

**Authors:** Diogo Martins, Michael A. DiCandia, Aristides L. Mendes, Daniela Wetzel, Shonna M. McBride, Adriano O. Henriques, Mónica Serrano

**Affiliations:** 1 Instituto de Tecnologia Química E Biológica António Xavier, Avenida da República, 2780-157 Oeiras, Portugal; 2 Department of Microbiology and Immunology, Emory University School of Medicine, Atlanta, GA USA

**Keywords:** Microbiology, Bacteria, Microbial genetics, Pathogens

## Abstract

Bacteria that reside in the gastrointestinal tract of healthy humans are essential for our health, sustenance and well-being. About 50–60% of those bacteria have the ability to produce resilient spores that are important for the life cycle in the gut and for host-to-host transmission. A genomic signature for sporulation in the human intestine was recently described, which spans both commensals and pathogens such as *Clostridioides difficile* and contains several genes of unknown function. We report on the characterization of a signature gene, *CD25890*, which, as we show is involved in the control of sporulation initiation in *C. difficile* under certain nutritional conditions. Spo0A is the main regulatory protein controlling entry into sporulation and we show that an in-frame deletion of *CD25890* results in increased expression of *spo0A* per cell and increased sporulation. The effect of CD25890 on *spo0A* is likely indirect and mediated through repression of the *sinRR*´ operon. Deletion of the *CD25890* gene, however, does not alter the expression of the genes coding for the cytotoxins or the genes involved in biofilm formation. Our results suggest that CD25890 acts to modulate sporulation in response to the nutrients present in the environment.

## Introduction

Many members of the Firmicutes phylum produce endospores (hereafter spores for simplicity), as resilient and metabolically dormant structures that can remain viable for long periods of time. For anaerobic bacteria, the production of oxygen-resistant spores is an important mechanism for transmission between hosts and persistence inside and outside the host^1^. A large proportion of recently identified members of the human gut microbiota are spore formers, many of which are anaerobic^1,2^ and in rare cases can cause opportunistic infections^1,3^. Other spore formers, however, have evolved to become dedicated pathogens that can cause a variety of diseases for which the transmissible and infectious agent is often the spore. Toxins are produced by the actively growing cells that result from spore germination, highlighting the central role sporulation has in the pathophysiology of infection^4^. One example is *Clostridioides difficile*, an obligate anaerobic nosocomial pathogen and the major causative agent of a range of intestinal diseases associated with antibiotic therapy in adults^5,6^.

Entry into sporulation requires phosphorylation of the Spo0A response regulator. Spo0A is conserved amongst spore formers and is essential for sporulation initiation in all organisms in which its function has been experimentally assessed^7–9^. The activation and role of Spo0A in sporulation has been studied in detail in *Bacillus subtilis*^10–14^. Spo0A is activated via phosphorylation by a phosphorelay consisting of several sensor kinases and by the phosphortransfer proteins Spo0F and Spo0B. The kinases respond to external signals by auto-phosphorylating an histidine residue and subsequently transferring a phosphoryl group via the intermediary proteins Spo0F and Spo0B to Spo0A^15^. The phosphorelay provides multiple points of control of the flow of phosphate to Spo0A and serves to integrate several signals, including metabolic or from the cell cycle, that promote entry into sporulation under conditions of extreme nutrient deprivation in a cell density-dependent manner^16^. In contrast to *B. subtilis*, no Spo0F or Spo0B homologs are present in *C. difficile*; rather, Spo0A is believed to be phosphorylated directly by orphan histidine kinases that respond to unidentified signals^17,18^. The *C. difficile* genome codes for five predicted orphan kinases, three of which, CD14920, CD15790 and CD24920, share sequence similarity with the phosphorelay sensor kinases of *B. subtilis*^18^. These three kinases were initially proposed to be responsible for Spo0A phosphorylation, but only CD15790 was shown to directly phosphorylate Spo0A. While the role of CD24920 is still unclear^18^, CD14920 is known to inhibit sporulation initiation and to affect toxin and motility through the regulatory proteins RstA and SigD^19^.

Although our knowledge of the regulatory network that controls initiation of sporulation is still incomplete, evidence suggests that *C. difficile* may directly respond to the nutritional potential of the environment^20^. Two global regulators, CcpA and CodY, involved in nutrient sensing, downregulate sporulation by repressing the expression of genes required for sporulation under high-nutrient conditions^21–24^. When nutrients are abundant, CodY binds branched chain amino acids and GTP and acts primarily as a transcriptional repressor of alternative metabolic pathways^22–24^. CcpA in turn governs the global response to carbon availability^21^. Both regulators repress expression of the *sinRR’* operon, which stimulates *spo0A* transcription and/or Spo0A activity^25^. The genome of *C. difficile* also encodes homologues of the *B. subtilis* Opp and App oligopeptide permeases, which in the latter organism are required for sporulation initiation^26,27^. In *C. difficile*, however, deletion of *opp/app* results in increased expression of the *sinRR’* operon and increased sporulation^20^. Presumably, peptide uptake increases the intracellular availability of amino acids and the activity of CodY and CcpA^20^.

In *C. difficile*, Spo0A controls approximately 300 genes^13,28^, which have been linked to biofilm formation, swimming motility, toxin production and sporulation^13,18,29^. Among the latter, are the genes coding for the first cell type-specific regulators of sporulation (σ^F^ and σ^E^) as well as genes required for asymmetric division^13,16,30–32^.

We previously established a genomic signature for sporulation, defined as the genes found in 90% of the endospore forming bacteria and present in no more than 10% of the non-endospore formers (^7^; Fig. S1A). This signature not only allows spore formation to be predicted for organisms for which sporulation has not been demonstrated in the laboratory, but also allows prediction of new genes potentially involved in the process^7^. More recently, a genomic signature for sporulation in the human gastro-intestinal tract was established (^1^; Fig. S1A). This signature includes 65 genes and is dominated by genes with a known function in sporulation. Approximately 30% of the signature genes, however, have no known function in sporulation and/or code for products with no similarity to known proteins^1^. Nevertheless, their presence in a genomic signature for sporulation suggests a role in spore development, at least in the gut. We show, that deletion of one of these genes, *CD25890*, increases sporulation as well as the expression of *spo0A* per cell, under certain nutritional conditions. We further show that the increased sporulation in the *CD25890* mutant is associated with the differential expression of the *sinRR’* operon, which was shown to be involved in sporulation, toxin production and motility. Deletion of the *CD25890* gene, however, does not alter the production of the cytotoxin TcdA or biofilm formation. Together our results suggest that CD25890 negatively modulates sporulation under certain nutritional conditions.

## Results

### Deletion of the *CD25890* gene

**Figure 1:**
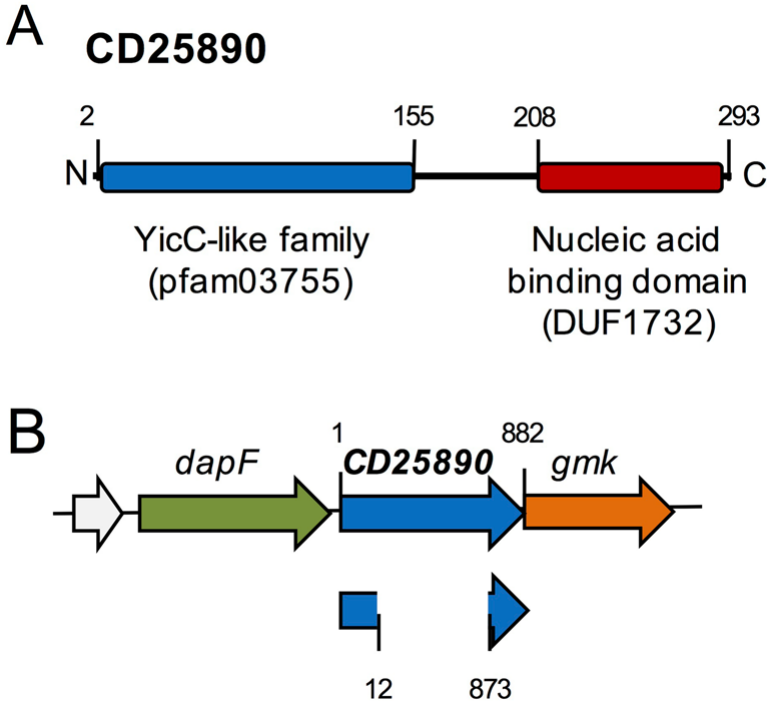
Sporulation and the CD25890 protein. (**A**) Schematic representation of the organization predicted for the CD25890 protein. CD25890 belongs to the YicC-like family of proteins (residues 2–155) and at the C-terminal has a domain that is predicted to bind to nucleic acids (residues 208–293). (**B**) schematic representation of the *CD25890* (CD25890) region of the *C. difficile* 630∆*erm* chromosome. The $\Delta$*CD25890* in-frame deletion removed codons 5–291 of the 293-codons.

Although present in a genomic signature for endosporulation, the *CD25890* gene has a broader distribution among eubacteria, and is found even in non-sporulating organisms (STRING database;^33^). For all Firmicutes genomes we examined, *CD25890* is found immediately upstream of the *gmk* gene coding for a guanylate kinase, as in *C. difficile*, or of the *remA* gene encoding a biofilm regulator (^34^; Fig.S1B). These genes, in turn, are always upstream of the *rpoZ* gene, coding for the omega subunit of RNA polymerase. Although its genomic context is conserved, there is no evidence for the possible function of *CD25890*. CD25890 belongs to the YicC-like family which includes poorly characterized proteins that play a role in survival during stationary phase (^35^; Fig. 1A). These proteins have a C-terminal domain of unknown function (DUF1732). Structural modelling of this domain suggests that it may bind to nucleic acids (^36^; Fig. 1A).

To study the role of CD25890 in the physiology of *C. difficile*, we inactivated the *CD25890* gene. An in-frame deletion mutant of *CD25890,* lacking codons 5–291 of the 293-codon open reading frame was generated by allelic-coupled exchange (ACE) in the background of the widely used 630$\Delta$*erm* strain (Fig. 1B; Fig. S2A and B; Table S5;^37^). The *CD25890* deletion mutation did not affect the expression of the downstream gene *gmk*, as evidenced by qRT-PCR analysis (Fig. S2C). To allow complementation analysis, a single copy of *CD25890* was inserted into the genome by ACE, upon restoration of the *pyrE* gene (Fig. S2A and B;^37^). In this strain, termed *CD25890*^c^, expression of the *CD25890* gene is restored, although to levels ~ threefold lower than in the wild-type (Fig. S2C). The lower expression of *CD25890* in the complemented strain may indicate that the gene is part of a large operon. In fact, previous work using genome-wide transcriptional start site (TSS) mapping, identified a unique TSS in this region, located 23 bp upstream of *dapF* (Fig. 1B). This suggests that *dapF* is the first gene of a large operon which includes CD25890^38^*.*

### Impact of *CD25890* deletion on biofilm formation, toxin production and sporulation

Since CD25890 belongs to the YicC-like family, and this family was reported to be involved in survival during stationary phase^35^, we started by determining whether the *CD25890* mutation had an impact on toxin production, biofilm formation or sporulation, all of which are stationary phase processes. We first tested the ability of these strains to form a biofilm in the presence of glucose or glucose with deoxycholate (DOC), using the crystal violet assay^39^. We found no effect of the *CD25890* mutation on the induction of biofilm formation in the presence of glucose alone or glucose with DOC after 24 h of growth (Fig. S3A).

We then used immunoblot analysis to investigate the levels of toxin (TcdA). The wild-type and the mutant strain were grown in TY, a medium that supports efficient toxin production^40^. Samples were collected 8, 10, 12 and 14 h after inoculation, and the presence of TcdA was monitored in the cell fractions. TcdA levels were similar in the wild type and *CD25890* mutant strain (Fig. S3B).

For sporulation, we examined cultures of the *CD25890* mutant after 14 h of incubation in sporulation liquid medium (SM). SM has been shown to induce sporulation in strain 630Δ*erm* and is well suited for kinetic analyses and fluorescence microscopy^41,42^. The cells were stained with the lipophilic membrane dye FM4-64, which allows identification of the stages of sporulation up to engulfment completion, prior to imaging by phase-contrast and fluorescence microscopy^41^. As shown in Fig. S3C, the *CD25890* mutant presents more cells with signs of sporulation than the wild-type strain or the complementation strain as determined by fluorescence and phase-contrast microscopy (630 $\Delta$*erm*, 29%; $\Delta$*CD25890*, 69%; *CD25890*^C^, 25%).

To rule out that the increased sporulation phenotype resulted from differences during vegetative growth, the wild-type and the *CD25890* mutant strains were grown in SM and the optical density of the cultures was measured at 600 nm (OD_600_) at two-hour intervals for 20 h after inoculation. Both strains showed similar growth rates (wild-type 0.658 and $\Delta$*CD25890* 0.613) and, after about 10 h, both entered stationary phase (Fig. S3D). Therefore, the *CD25890* mutation does not impact the growth of *C. difficile* under the conditions tested.

### Disruption of *CD25890* results in increased spore formation

**Figure 2:**
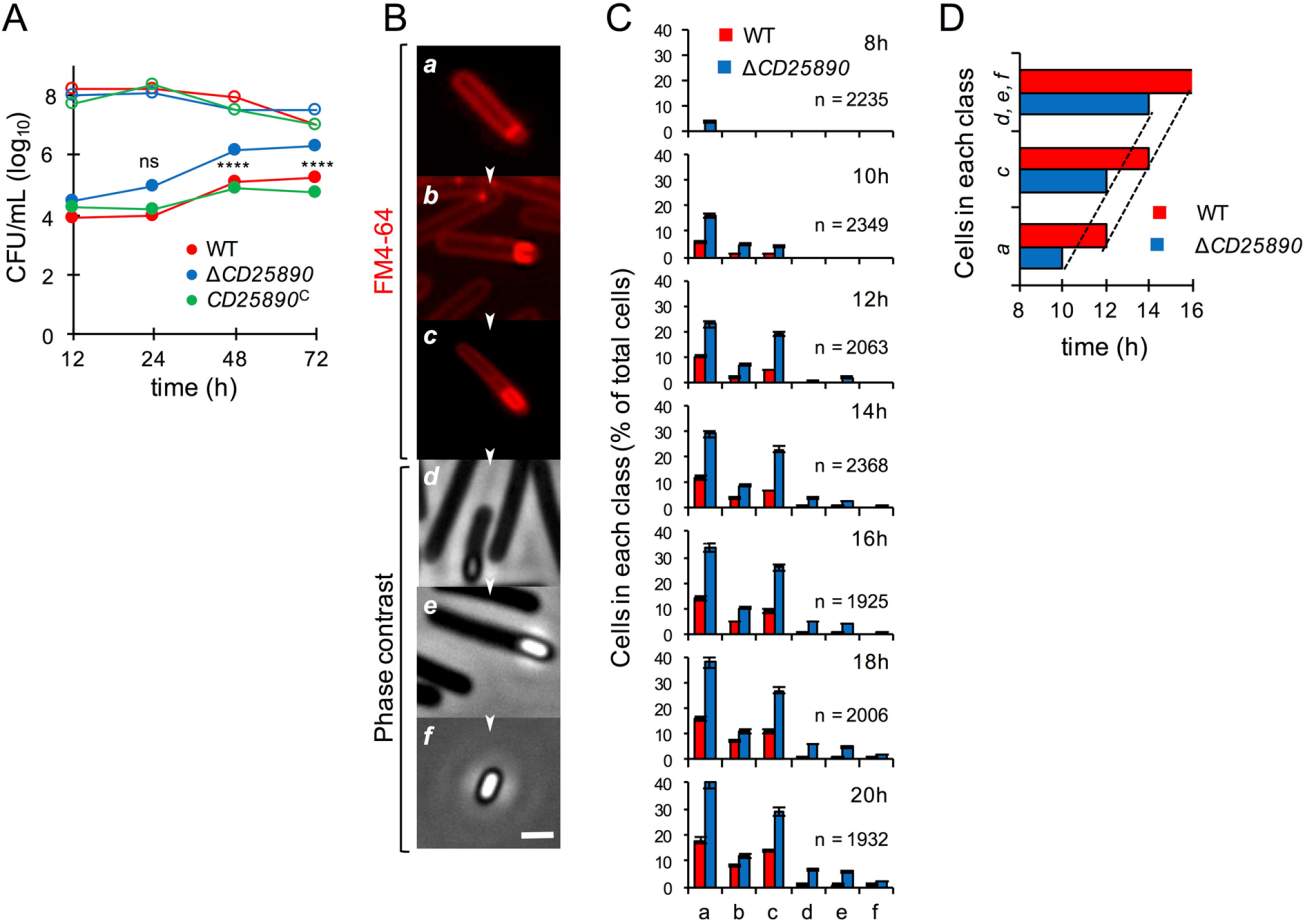
Analysis of $\Delta$*CD25890* mutant sporulation dynamics. (**A**) Cells were grown in liquid SM and the titer of heat resistant spores (filled circles) and total viable cells (open circles) measured 12, 24, 48 and 72 h following inoculation. WT, red; $\Delta$*CD25890* mutant, blue; *CD25890*^*C*^ (complementation strain), green. The date in the graph represent the mean of three independent experiments (see also Table S1). Asterisks indicate statistical significance determined with a two-way ANOVA (ns, no significant; **** p < 0.0001). (**B**) The panels (a-f) illustrate the sequence of the sporulation stages quantified in (**C**) the process begins with an asymmetric cell division (**a**), then, the mother cell membrane migrates around the forespore engulfing it (b). At the end of this process, the forespore becomes a free protoplast in the mother cell cytoplasm (c). Finally, the spore protective layers are synthesized and deposited around the developing spore (d and e). Upon mother cell lysis, a mature spore is released to the environment, where it remains in a dormant state until germination-promoting conditions are met (f). Images were acquired by Metamorph™ (version 5.8; Molecular Devices) (**C**) Samples of an SM liquid culture of the WT strain were collected at 8, 10, 12, 14, 16, 18 and 20 h after inoculation, stained with the membrane dye FM4-64 and examined by phase-contrast or fluorescent microscopy. Quantification of the percentage of cells in the morphological classes represented in B (*a* to *f*), relative to the total viable cell population, for the WT (red) and $\Delta$*CD25890* mutant (blue) at the indicated times following inoculation in liquid SM. The data represent the mean ± SD of three independent experiments. The total number of cells scored (n) is indicated in each panel. (**D**) Time, in hours, when at least 10% of WT or $\Delta$*CD25890* population reached the indicated stages of sporulation. The top columns show phase-bright, phase-grey spores or free spores (morphological classes *d*, *e* and *f*), the middle columns engulfment completion (*c*), and the bottom columns asymmetric septation (*a*).

To determine more precisely whether the *CD25890* mutant sporulated more efficiently than the wild-type, the *CD25890* mutant, wild-type, and the *CD25890*^c^ complement strain were analysed for their ability to form heat resistant spores. Strains were grown in SM broth, and at 12, 24, 48 and 72 h the titer of heat resistant spores/ml of culture was determined as described in the Materials and Methods section (Fig. 2A and Table S1). In line with earlier results^41^, the titer of spores for the wild-type strain increased from 7.5 × 10^3^ spores/ml at hour 12, to 1.0 × 10^4^ spores/ml at hour 24, 1.3 × 10^5^ spores/ml at hour 48 and 1.7 × 10^5^ spores/ml at hour 72 (Fig. 2A and Table S1). As expected from the previous results, the *CD25890* mutation did not affect cell viability (Table S1). In contrast, the titer of heat resistant spores in the mutant strain was higher than the wild-type for all time points: 3.1 × 10^4^, 8.5 × 10^4^ spores/mL, 1.5 × 10^6^ and 2.2 × 10^6^ spores/mL at hour 12, 24, 48 and 72, respectively (Fig. 2A and Table S1). The complemented *CD25890*^c^ strain restored the wild-type kinetics of spore formation (1.8 × 10^4^, 1.5 × 10^4^, 7.8 × 10^4^ and 6.0 × 10^4^ spores/ml at hour 12, 24, 48 and 72, respectively; Fig. 2A and Table S1). Thus, deletion of *CD25890* is responsible for the increased sporulation frequency of the mutant.

Another frequently used method to examine sporulation is growth on agar plates of 70:30 medium, in which the rate of sporulation is higher than in the SM broth used above^43^. We tested the sporulation efficiency of the *CD25890* mutant in this medium. When sporulation was induced on 70:30 plates, the *CD25890* mutant showed a percentage of sporulation similar to the wild-type strain (Fig. S4A). Thus, the culture medium influences the *CD25890* mutant phenotype (see also below).

We next followed the morphological stages of sporulation in SM medium by phase-contrast and fluorescence microscopy^41^. The wild-type and the mutant strain were grown in SM and samples were collected 8, 10, 12, 14, 16, 18 and 20 h after inoculation. The cells were stained with FM4-64 prior to microscopic examination. Cells representative of several distinctive morphological classes are shown on Fig. 2B. We observed that 8 h after inoculation, a significant fraction of the *CD25890* cells already processed polar division (Fig. 2C; class *a*). In contrast, class *a* cells were detected for the wild-type strain 2 h later than in the mutant. Moreover, phase bright spores (class *e*) were observed at 14 h for the mutant and at 18 h for the wild-type strain (Fig. 2C).

To test whether spore morphogenesis proceeded faster in the *CD25890* mutant, we determined the time at which at least 10% of the cell population completed polar division (class *a*), initiated engulfment (class *b*), showed complete engulfment of the forespore by the mother cell (class *c*), or presented phase-gray or phase-bright spores (classes *d* and *f*). The results in Fig. 2D show that progress through these stages of sporulation occurred at the same pace for both strains. We conclude that the *CD25890* mutant initiated sporulation earlier than the parental wild-type or that the fraction of cells entering sporulation at the end of growth is greater for the mutant. In any event, CD25890 negatively influenced the entry into sporulation.

### CD25890 accumulates during growth independently of Spo0A

**Figure 3:**
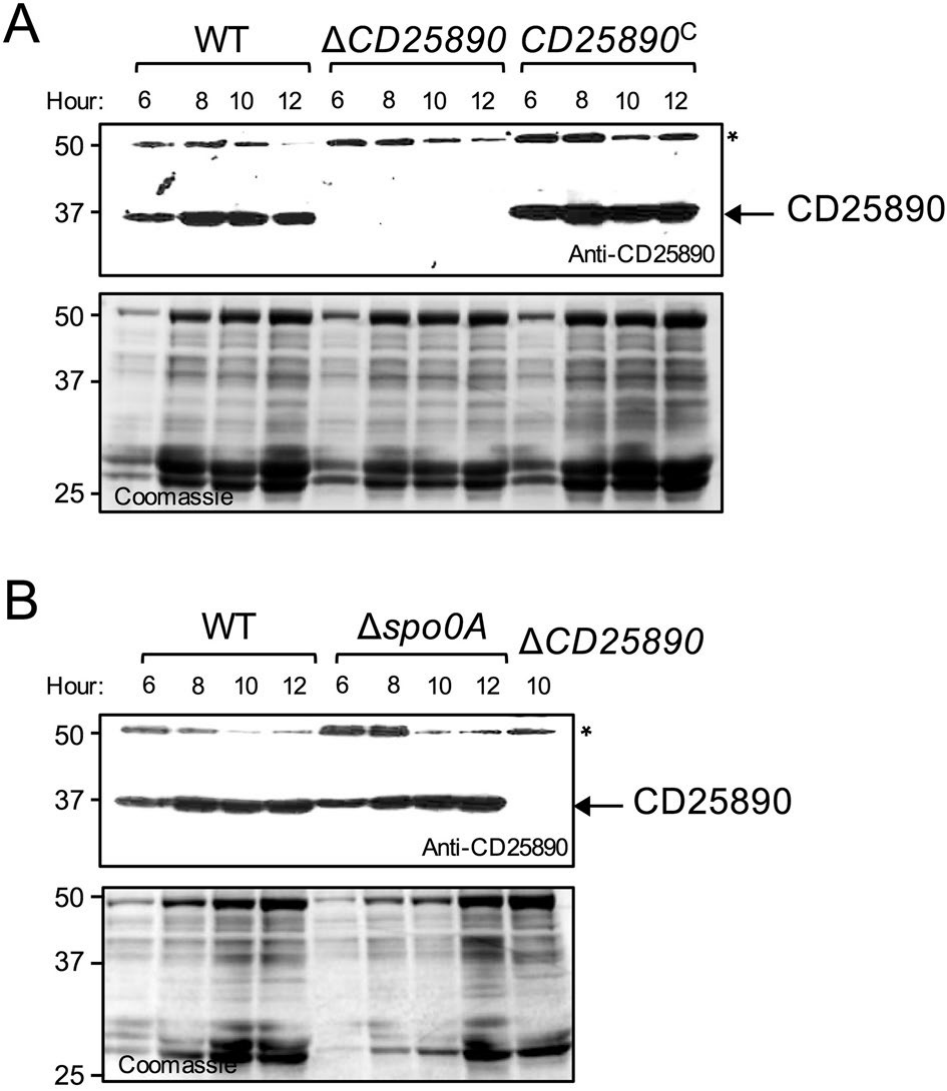
Accumulation of CD25890 during growth. The wild type strain (WT), the $\Delta$*CD25890* mutant, the complementation strain (*CD25890*^C^) (**A**) and the *spo0A* mutant (**B**) were grown in SM and samples were collected at 6, 8, 10 and 12 h after inoculation for western blot analysis using anti-CD25890 antibody (* cross-reactive species). The position of molecular weight markers (in kDa) is indicated on the left side of the panels.

We further investigated accumulation of CD25890 during growth and sporulation in SM broth by immunoblot analysis using an anti-CD25890 antibody (Fig. 3A). CD25890 started to accumulate early during growth and was present during entry into stationary phase when the cells start to sporulate. No signal was detected in the *CD25890* mutant, while its accumulation was restored in the complementation strain, confirming the specificity of the antibody. CD25890 accumulation was independent of Spo0A (Fig. 3B). Thus, CD25890 starts to accumulate during vegetative growth, independently of the main transcriptional regulator for entry into sporulation, Spo0A.

### Deletion of *CD25890* results in increased accumulation of Spo0A

**Figure 4:**
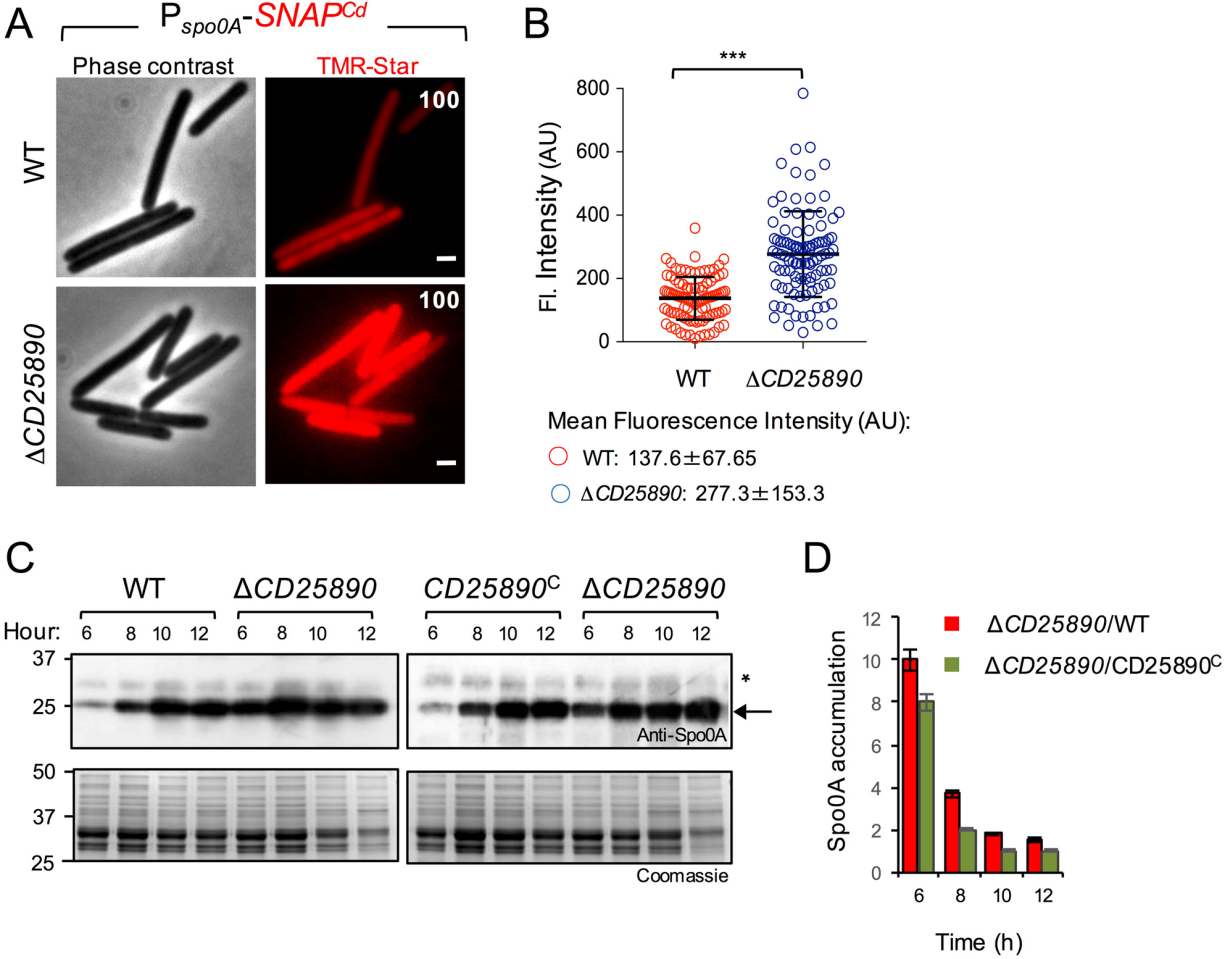
Increased expression of *spo0A* in the $\Delta$*CD25890* mutant. A: Microscopy analysis of *C. difficile* cells carrying a P_*spoOA*_-*SNAP*^*Cd*^ transcriptional fusion in the WT and congenic $\Delta$*CD25890* mutant. The cells were collected after 10 h of growth in SM broth, stained with TMR-Star, and examined by fluorescence microscopy to monitor SNAP production. The panels are representative of the expression patterns observed. The numbers refer to the percentage of cells showing SNAP fluorescence. Data shown are from one experiment and are representative of at least three independent experiments. Scale bar, 1 µm. B: Quantitative analysis of the fluorescence intensity (Fl.) for single cells with no signs of sporulation (n = 100 cells) of the two strains in A. Data shown are from one experiment, representative of at least three independent experiments. The numbers in the legend represent the mean and the SD of fluorescence intensity. ***, p < 0.001. Images were acquired by Metamorph™ (version 5.8; Molecular Devices) C: Samples were collected from the wild type strain (WT), the $\Delta$*CD25890* mutant and the complementation strain (*CD25890*^C^) grown in liquid SM, at the indicated times after inoculation. Extracts were prepared and proteins (15 µg) resolved by SDS-PAGE and subjected to immunobloting using an anti-Spo0A antibody. The position of molecular weight markers (in kDa) is indicated on the left side of the panels, and the arrow on the right indicates the position of Spo0A. D: Spo0A accumulation was assessed by quantification of the intensity of bands in the immunoblots using the Image J software and is shown as the ratio between the $\Delta$*CD25890* mutant and the WT (red bars) or the complementation strain (green bars) at the indicated times (in hours) after inoculation in liquid SM. All data represent the mean ± SD from three independent experiments.

The role of Spo0A in governing entry into sporulation in *C. difficile* has been established^13,18,32^. We wanted to test if deletion of *CD25890* increased expression of the *spo0A* gene. We constructed a fusion of the *spo0A* promotor region to the *SNAP*^*Cd*^ reporter and we introduced the P_*spo0A*_*-SNAP*^*Cd*^ fusion in the *CD25890* mutant and in the parental wild-type strain (Table S5). To monitor production of the *SNAP*^*Cd*^ reporter, samples of cultures bearing the transcriptional fusion were collected 10 h after inoculation in SM and the cells were labelled with TMR-Star (Fig. 4A). SNAP production from P_*spo0A*_*-SNAP*^*Cd*^ was detected in 100% of the cells for both the wild-type and the *CD25890* mutant (Fig. 4A). Quantification of the fluorescence signal per cell (in arbitrary units, or AU; Fig. 4B), however, shows that transcription from the *spo0A* promoter occurred at a lower intensity in the wild-type (average signal, 137.6 arbitrary units, AU) compared to the *CD25890* mutant (average signal, 277.3 AU) (Fig. 4B).

To investigate whether the increased transcription from the *spo0A* promoter could also lead to increased accumulation of Spo0A, we compared the levels of Spo0A throughout sporulation by immunoblot analysis using a previously described anti-Spo0A antibody (^44^; Fig. 4C). We detected Spo0A as early as 6 h following inoculation in SM for all strains. Quantification of band intensity however, shows that in the *CD25890* mutant Spo0A accumulated at higher levels than in the wild-type or in the complementation strain at hours 6 (8 to 10 times more) and 8 (4 to 2 times more) (Fig. 4C,D). Together, these results suggest that in the mutant, more cells reach a threshold level of Spo0A that triggers sporulation. Moreover, since *spo0A* is subject to positive auto-regulation^32^, the results also suggest that CD25890 antagonizes the expression of *spo0A* and/or the activity of Spo0A.

Since we did not observe a phenotype for the *CD25890* mutant when sporulation was induced on 70:30 medium (see above), we compared the expression of *spo0A* in SM broth to 70:30 medium using fluorescence microscopy and single cell analysis (Fig. S4B). Quantification of the fluorescence signal per cell (Fig. S4B) showed that transcription from the *spo0A* promoter was higher on 70:30 medium in both the wild-type (average signal, 267.1 AU) and *CD25890* mutant (average signal, 332.8 AU), reaching levels similar to those observed in the mutant when sporulation was induced in SM (average signal, 372 AU) (Fig. S4B). This result suggests that on 70:30 plates, the wild-type strain reaches the threshold levels of *spo0A* required for sporulation in a higher number of cells than in SM broth, similar to what was observed for the *CD25890* mutant in SM. Hence, under certain nutritional conditions, CD25890 acts to curtail sporulation initiation.

### Phosphorylated Spo0A accumulates at higher levels in the *CD25890* mutant

**Figure 5:**
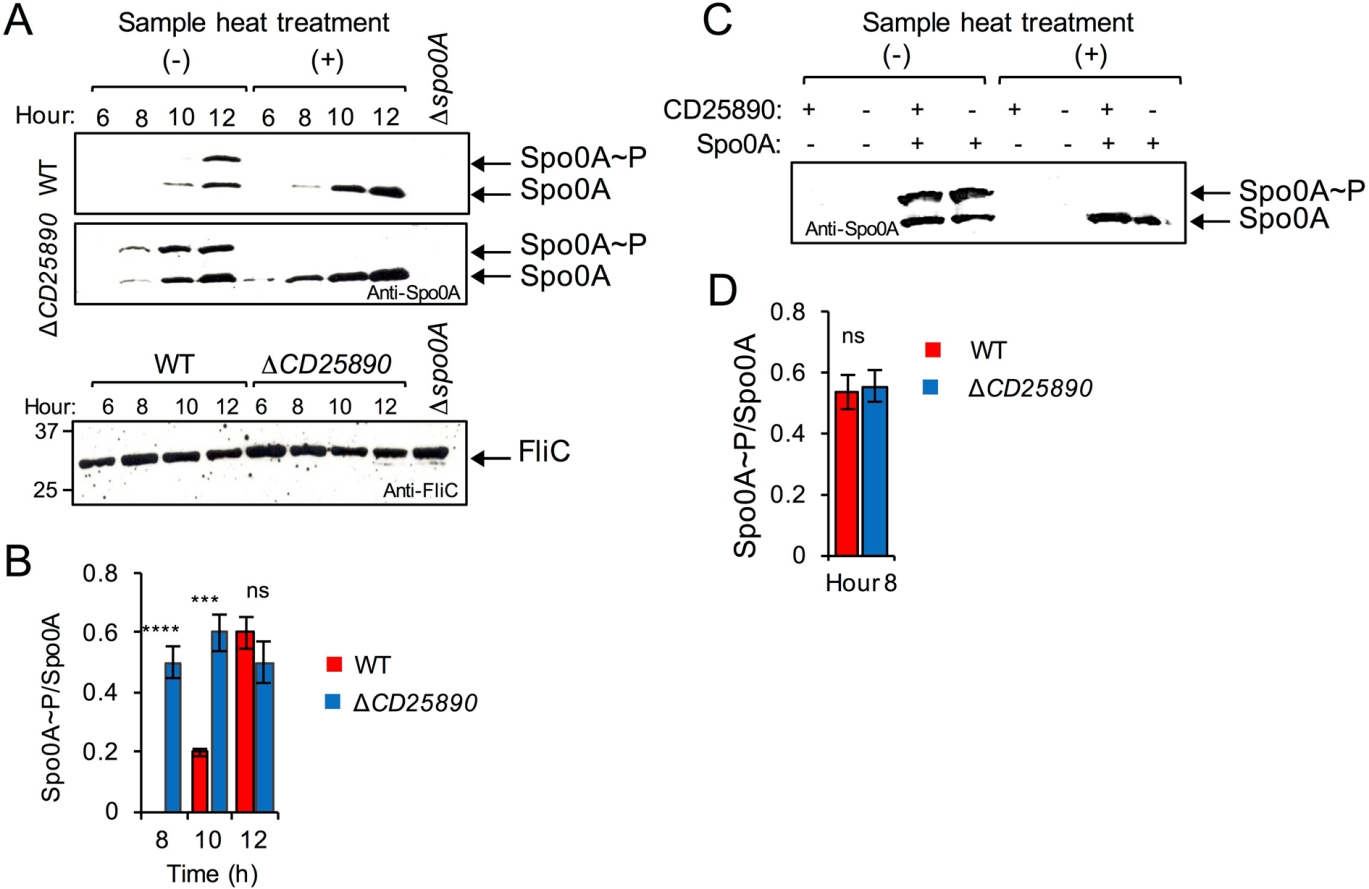
Phosphorylation of Spo0A in the $\Delta$*CD25890* mutant. (**A**) Samples were collected from the WT and the $\Delta$*CD25890* mutant grown in liquid SM, at the indicated times after inoculation. Extracts were prepared and proteins (15 µg) resolved by Phos-tag SDS-PAGE and subjected to immunobloting using an anti-Spo0A antibody. In the bottom panel the same extracts were loaded in a SDS-PAGE and subjected to immunoblotting using an anti- FliC antibody, as a loading control. The position of molecular weight markers (in kDa) is indicated on the left side of the panels. (**B**) and (**D**): The ratio of Spo0A ~ P to Spo0A was assessed with Image J software and represented for the WT and the $\Delta$*CD25890* mutant at the indicated times (in hours) after inoculation in liquid SM. All data represent the means ± SD from three independent experiments. Asterisks indicate statistical significance determined by two-tailed Student t-test (ns, no significant; **** p < 0.0001, *** p < 0.001). C: Extracts were prepared from liquid SM cultures of the P_*tet*_-*spo0A* and $\Delta$*CD25890* P_*tet*_-*spo0A* strains grown in the presence (50 nM) or in the absence of anhydrotetracycline, 8 h after inoculation. Proteins (15 µg) were resolved by Phos-tag SDS-PAGE and subject to immunoblotting using an anti-Spo0A antibody. In A and (**C**) The faster migrating bands (black arrows) show the unphosphorylated form of Spo0A (Spo0A), and the slower migrating bands indicate the phosphorylated form of Spo0A (Spo0A ~ P). The samples heated at 100 °C for 5 min were loaded as a control for the position of unphosphorylated Spo0A.

Spo0A is activated by phosphorylation^45,46^. The activated Spo0A ~ P, binds to DNA promoter regions containing a Spo0A-binding motif and regulates the expression of Spo0A-dependent genes. Since *spo0A* is auto-regulatory, and higher expression per cell was seen in the *CD25890* mutant in SM, we asked whether Spo0A ~ P would accumulate at higher levels in the mutant. We used Phos-tag SDS-PAGE, where migration of Spo0A ~ P is delayed, and the two forms (phosphorylated and unphosphorylated) are detected by western blot using anti-Spo0A antibodies. The wild-type and the mutant strain were grown in SM and samples were collected 6, 8, 10 and 12 h after inoculation. Whole cell extracts were prepared and the proteins resolved by Phos-tag SDS-PAGE (Fig. 5A). Two bands were detected in this manner. The upper band disappeared when the samples were boiled prior to electrophoretic resolution, which indicates that the upper band corresponds to Spo0A ~ P and the lower band to the unphosphorylated form^47^. In the *CD25890* mutant, both forms of Spo0A accumulate earlier and at higher levels than in the wild-type (Fig. 5A). We then quantified band intensities in order to estimate the ratio of the phosphorylated to unphosphorylated form of Spo0A (Spo0A∼P/Spo0A). The Spo0A∼P/Spo0A ratio was higher at hour 8 and 10 in the mutant as compared to the wild-type strain (Fig. 5B). At hour 12, however, the ratio between the two forms was similar between the two strains.

Together with the observation that *spo0A* is expressed in most if not all of the pre-divisional cells (Fig. 4A), this result suggests that the level of Spo0A phosphorylation correlates with the level of the protein. Ultimately, the level of *spo0A* per cell may limit entry into sporulation.

### Deletion of *CD25890* increases transcription of *spo0A*

Since there is a positive feedback loop in which Spo0A∼P controls *spo0A* transcription^32^, we cannot discriminate if the increased sporulation in the mutant could be due to increased transcription or to increased activity of Spo0A. To uncouple transcription from activation of Spo0A by phosphorylation, we placed the *spo0A* gene under the control of the anhydrotetracycline (ATc) responsive P_*tet*_ promoter^48^. This experimental setup allows Spo0A to accumulate to similar levels in the wild-type and in the *CD25890* mutant strain, and to test whether Spo0A ~ P accumulated to a higher level in the mutant. The strains were grown in SM supplemented with 50 nM of ATc, a concentration at which Spo0A accumulates to levels similar to the wild-type strain^48^, and proteins in whole cells extracts were resolved by Phos-tag SDS-PAGE (Fig. 5C). In both strains Spo0A accumulated at similar levels and the Spo0A ~ P/Spo0A ratio was similar (Fig. 5C,D). In agreement with this observation, we found no difference in the titer of heat resistant spores produced by both strains (Table S2).

These results suggest that CD25890 acts to decrease the levels of *spo0A* transcription per cell. Since CD25890 has a putative DNA-binding domain (DUF 1732; Fig. 1), this effect may be direct or indirect.

### Deletion of *CD25890* influences the expression of the *sinRR’* operon

**Figure 6:**
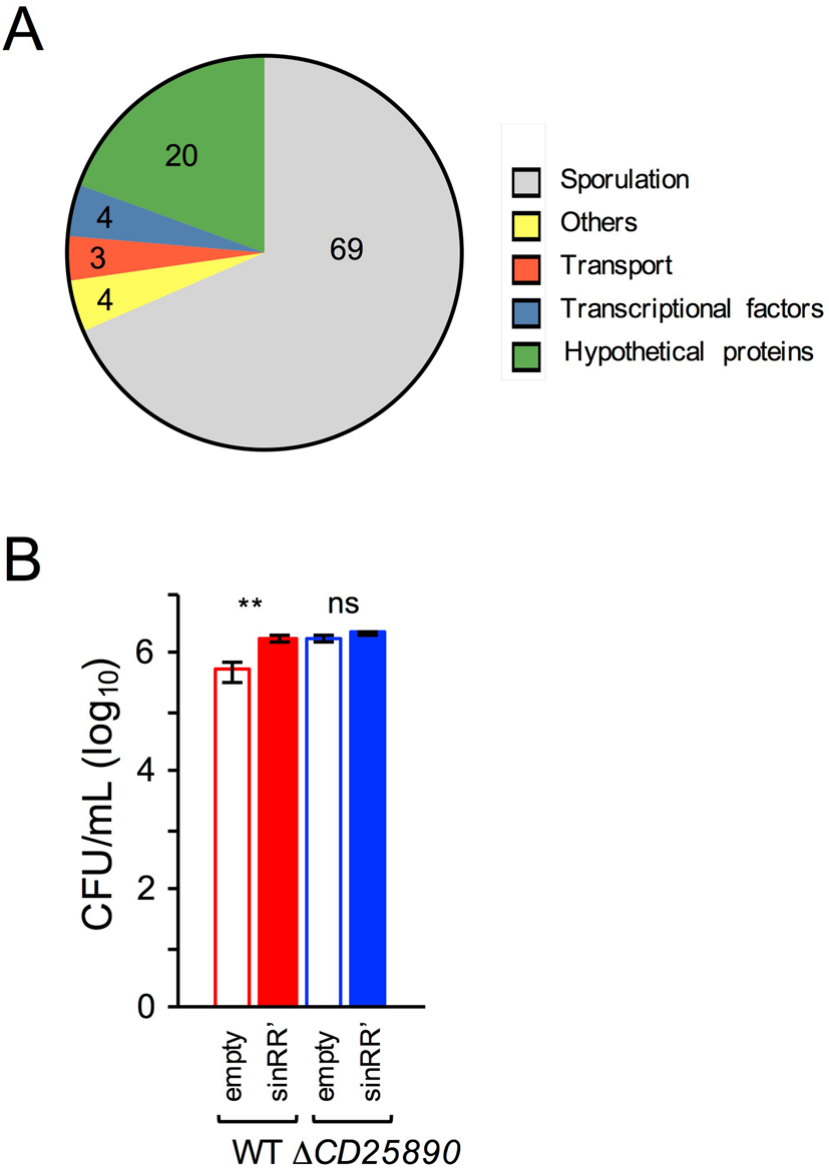
Differential gene expression in the $\Delta$*CD25890* mutant. (**A**) To compare the transcriptome of the $\Delta$*CD25890* mutant with the wild type, both strains were grown for 10 h in SM broth, samples collected and RNA-seq performed. The graph represents the functional classes of the 165 genes that are up-regulated in the $\Delta$*CD25890* mutant based on the RNA seq data (the number represent the percentage of each functional class). See also Table S3. (**B**) Sporulation efficiency of the P*tet*-*sinRR’* alleles. The spore titer was determined 24 h following inoculation into SM sporulation medium supplemented with 50 nM of anhydrotetracycline. The heat resistant spore count by determining the cfu/mL obtained after treatment at 70ºC. The results shown are averages and standard deviations for three biological replicates.

To determine whether the *CD25890* mutation had a more generalized effect on gene expression, we decided to compare the transcriptome of the mutant with the wild type after 10 h of growth in SM. We used two biological replicates and genes were considered differentially expressed if the fold change was > 2 and the adjusted p value < 0.01. 165 genes were overexpressed in the mutant, while only 21 genes were down-regulated (Table S3). From the upregulated genes, 69% of genes are regulated by Spo0A or by sporulation-specific cell type-specific sigma factors (Fig. 6A and Table S3). In contrast, no sporulation genes were found among the downregulated genes (Table S3). No toxin, biofilm or motility genes were among the genes differentially expressed in the *CD25890* mutant (Table S3). However, the *sinRR’* operon showed increased expression in the *CD25890* mutant (~ fivefold, relative to the wild type; Table S3). The *sinRR’* operon was previously shown to be involved in sporulation, toxin production and motility^25^. Overexpression of *sinR* increased sporulation efficiency, while overexpression of *sinR’* reduced sporulation^25^. To our knowledge, however, the effect on sporulation of overexpression of the entire operon was not tested. To test the effect of overexpression of the *sinRR’* operon, a plasmid containing the *sinRR’* operon under the tetracycline-inducible promoter was introduced into the wild-type strain and the *CD25890* mutant and sporulation was tested upon induction with ATc (Fig. 6B and Table S4). The same strains with the empty vector were used as a control in this assay. Sporulation assays were performed in bacterial cultures grown in SM supplemented with 50 ng/ml of ATc for 24 h. Overexpression of the *sinRR’* operon in a wild type strain increased sporulation efficiency ~ 3.4—fold when compared to the wild-type control strain (Fig. 6B and Table S4). A similar increase relative to the wild-type was observed in the *CD25890* mutant carrying the empty vector (~ 3.46 -fold). No significant effect on sporulation was observed when *sinRR’* was overexpressed in a *CD25890* mutant compared to the mutant control strain (Fig. 6B and Table S4). Therefore, overexpression of the *sinRR’* operon leads to increased sporulation efficiency. In the *CD25890* mutant background, however, overexpression of *sinRR’* did not have a cumulative effect on spore production, suggesting that both alleles may act in the same pathway, leading to increased *spo0A* transcription.

Among the genes whose expression is independent of Spo0A, but exhibit differential expression in the *CD25890* mutant, we identified putative transcriptional regulators that are upregulated in the mutant strain (*CD21430* and *CD24890*), or downregulated (*CD03700*) (Table S3). It is tempting to speculate that CD25890 could act on *spo0A* and *sinRR’* through the action of these Spo0A-independent regulators.

### Virulence and colonization of the ∆*CD25890* mutant in an animal model

**Figure 7:**
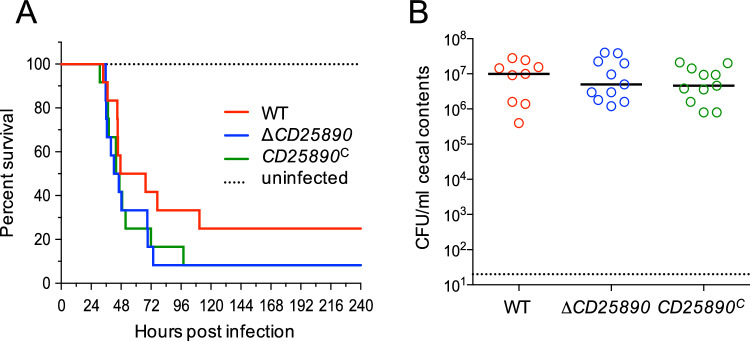
The $\Delta$*CD25890 mutant* show no differences in virulence. (**A**) and (**B**) Syrian golden hamsters were inoculated with approximately 5000 spores of strain 630∆*erm* (n = 12), *CD25890* (n = 12), or *CD25890*^*C*^ (n = 12). (**A**) Kaplan–Meier survival curve depicting time to morbidity. Mean times to morbidity were: 630∆*erm* 56.6 ± 8.2 (n = 9); *CD25890* 48.7 ± 4.5 (n = 11); *CD25890*^*C*^ 49.8 ± 5.8 (n = 11). (**B**) Total *C. difficile* CFU recovered from cecal contents collected post-mortem. Dotted line demarcates limit of detection. Solid black line marks the median. Numbers of CFU are compared to 630∆*erm* by one-way ANOVA with Dunnett’s multiple comparisons test. No statistically differences were observed.

Since the ∆*CD25890* mutant exhibited increased sporulation frequency in vitro, we decided to investigate the function of CD25890 in sporulation in the intestine and its effect on pathogenesis in the acute Golden Syrian hamster model of infection. Male and female Syrian golden hamsters were infected by oral gavage with approximately 5,000 spores of the parent strain 630 $\Delta$*erm*, the ∆*CD25890* mutant or the complemented strain, *CD25890*^*c*^. Following inoculation, animals were monitored for disease symptoms and fecal samples were collected every 24 h post-infection for enumeration of *C. difficile*. As shown in Fig. 7A, no significant difference was observed for the average time of post challenge survival between hamsters infected with the wild-type and the ∆*CD25890* mutant strain (wild-type 56.6 ± 8.2; ∆*CD25890* 48.7 ± 4.5). Moreover, *C. difficile* CFU counts were similar in the cecal contents of the ∆*CD25890* mutant and parental strain of infected animals post-mortem (Fig. 7B). These data indicate that despite the fact that the ∆*CD25890* mutant had a higher sporulation frequency in vitro, CD25890 has no statistically significant effect on the virulence in the infection model.

## Discussion

A genomic signature of sporulation in the human intestinal microbiome includes not only genes with an established function in sporulation (Fig. S1B) but also a group of 20 genes of unknown function^1^. We hypothesized that these uncharacterized genes may be important for sporulation. Here, we constructed an in-frame deletion mutation of one identified gene, *CD25890*, and characterized the resulting mutant. We show that the CD25890 protein accumulates during growth and stationary phase in SM medium in which sporulation is induced (Fig. 3). In agreement with our prediction, we found that under certain culturing conditions, deletion of *CD25890* impacts sporulation. The *CD25890* mutant reaches a higher titer of spores than the wild type strain when grown in sporulation medium (SM) and we show that in the mutant, spore differentiation is triggered in a larger fraction of the population (Fig. 2). The *CD25890* mutant maintains high levels of *spo0A* expression and shows higher expression of genes required for sporulation (Fig. 4 and Fig. 6 and Table S3). Since Spo0A is auto-regulatory, the transcriptional fusion reports both transcription of *spo0A* as well as the activity of Spo0A which is activated via phosphorylation^32^. We show that Spo0A ~ P accumulates to higher levels in the *CD25890* mutant earlier during growth*,* leading to increased expression of Spo0A-dependent genes. Uncoupling of transcription from activation by using an inducible promoter to control *spo0A* expression showed that CD25890 does not act on the phosphorylation status of Spo0A but rather at the level of *spo0A* transcription (Fig. 5).

Our finding that higher accumulation of Spo0A correlates with enhanced sporulation contrasts with studies performed in *B. subtilis*, where accelerated accumulation of Spo0A independent of the phosphorelay is detrimental to sporulation^49,50^. Rather, Spo0A needs to accumulate in a gradual manner to trigger sporulation and this requires its phosphorylation through the phosphorelay^49^. Thus, a gradual build-up of Spo0A ~ P may not be important for efficient sporulation in *C. difficile*. Previous studies have already shown that *spo0A* expression can be increased from an ATc-inducible promoter in a dose-dependent manner, and that the level of expression correlates with sporulation efficiency, which reaches values higher than those observed in a congenic wild-type strain^48^.

The impact of *CD25890* on sporulation and on the expression of sporulation-specific genes is seen in SM broth but not on 70:30 agar medium. Notably, SM is mainly rich in amino acids^42^. Interestingly, the ability of *C. difficile* to metabolize amino acids via Stickland fermentation appears important for the colonization of some perturbed communities^51,52^. An amino acid rich medium may permit *C. difficile* to grow and multiply, delaying entering in sporulation. It is tempting to suggest that *CD25890* would repress entry into sporulation in response to amino acid availability, but this remains to be tested (see also below). Conversely, 70:30 medium^43^ contains BHI, a more complex medium, possibly closer to the nutrient landscape of a healthy gut in which *C. difficile* is not able to proliferate; sporulation would then be a resort to persist within the host. As a strict anaerobe, *C. difficile* relies on spore formation for transmission between hosts but spores may also be a factor in persistence in the host and the immediate environment^10^. Sporulation appears to be an important function of the gut microbiota as about 60–70% of the organisms found in the gut are anaerobic spore formers^10^. Importantly, *B. subtilis* gastro-intestinal isolates tune the phosphorelay to favour sporulation, illustrating the importance of sporulation in the gut environment^44,53^.

**Figure 8:**
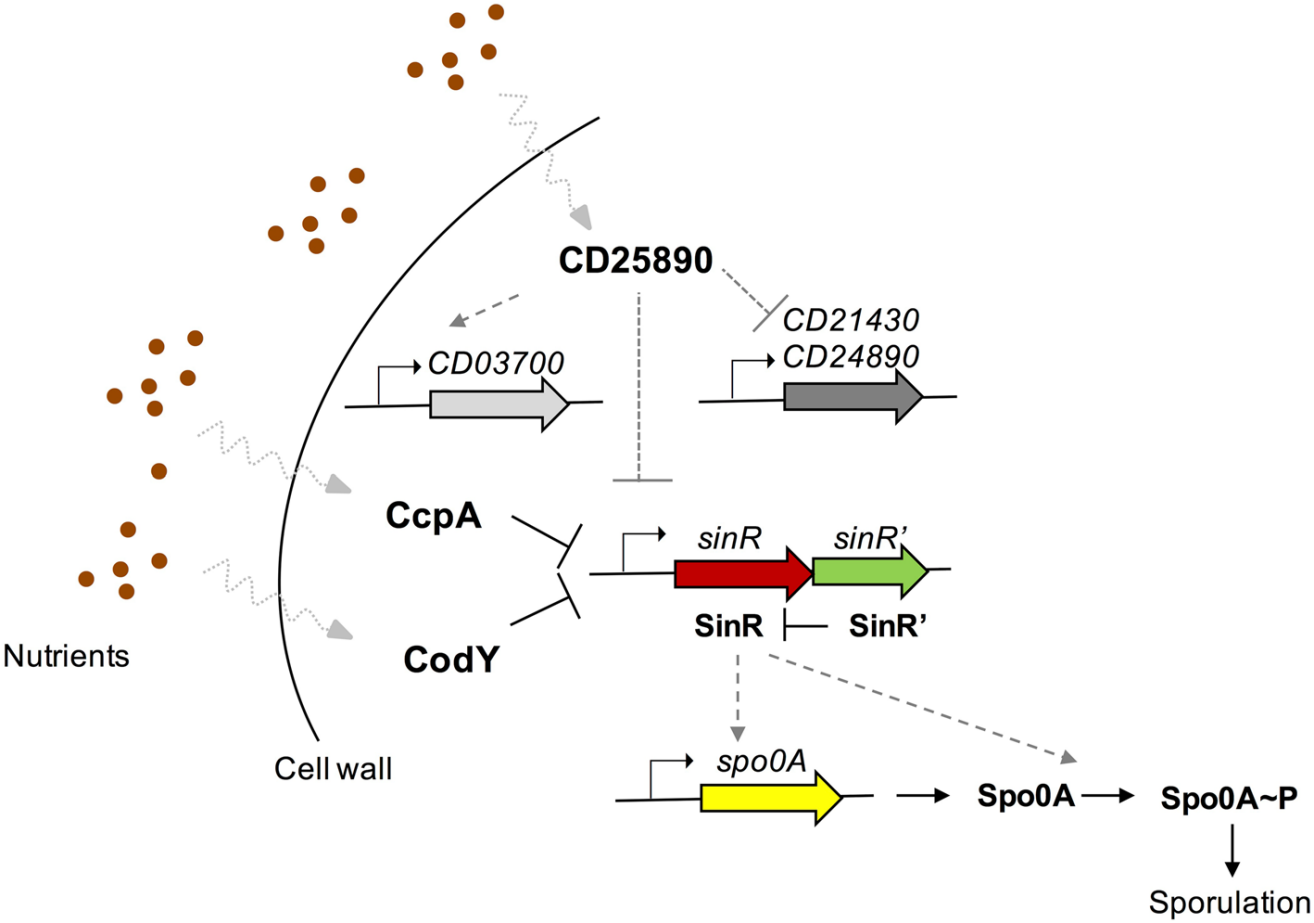
Model for the effect of the *CD25890* gene on sporulation initiation in *C. difficile*. CD25890 responds to the nutrient sources and indirectly influences the *sinRR’* operon expression through an unknown mechanism which represses sporulation-specific gene expression via *spo0A* expression. This mechanism may involve the transcriptional factors, CD03700, CD21430 and CD24890, which are differentially expressed in the *CD25890* mutant. CodY and CcpA are global regulators that also respond to nutrient cues and impact sporulation by directly repressing sinRR’ expression. Solid line, direct effect; dotted line, indirect effect.

Although our knowledge of the regulatory networks that control sporulation initiation in *C. difficile* is still incomplete, several lines of evidence suggest a direct link to the availability of nutrients in the environment. Two global regulators known to respond to nutrient availability in *C. difficile* are CodY and CcpA^21–23,25^. Both regulators affect the initiation of sporulation by repressing the expression of sporulation-related genes, such as the *sinRR’* operon, in response to glucose and branched chain amino acids, respectively^21–25^. In *C. difficile*, SinR has a positive effect on sporulation, perhaps by elevating the expression of *spo0A* by an unknown mechanism^25^. Accordingly, in strain 630, a *codY* mutant shows increased sporulation^24^. The activity of CodY may be regulated through the intracellular level of branched-chain amino acids, and the pools of these amino acids appear to be influenced by the oligopeptide permeases, Opp and App^24^. Disruption of *opp* and *app* results in increased sporulation-specific gene expression and a hyper-sporulation phenotype^20^. One possibility is that Opp and App function to import peptides that increase the pool of branched-chain amino acids, thereby activating CodY and leading to repression of *sinRR*´ and of sporulation^24^. In a striking parallel with the *opp*-*app* and *codY* mutants, disruption of the *CD25890* gene also results in increased sporulation-specific gene expression and a hyper-sporulation phenotype. Moreover, expression of the *sinRR’* operon is also elevated in the *CD25890* mutant (Table S3). It thus seems plausible that the hyper-sporulation phenotype seen in the *CD25890* mutant also stems, at least in part, from increased expression of *sinRR´* (Fig. 8)*.* It was previously observed that when expressed from its native promoter, *sinR’* transcripts levels were always reduced compared to *sinR* transcripts^25^. We suggest that when expression of the operon is increased, SinR levels are in greater abundance relative to SinR’, resulting in increased sporulation. It remains unknown whether *opp-app/codY* and *CD25890* function in the same or in different pathways that converge on *sinRR*´. However, while CodY and CcpA also repress the expression of toxin-encoding genes^21^, the expression of CodY and CcpA targets are not significantly changed in the *CD25890* mutant as determined by RNA-seq (Table S3). Thus, CodY and CcpA may not be involved in the same pathway as CD25890. In addition, *opp-app* mutants hyper-sporulate in 70:30 medium^20^, where disruption of *CD25890* has no effect on sporulation. More work is required to unravel the regulatory network involving *opp/app*, *codY*, *CD25890* and other factors that modulate sporulation initiation in *C. difficile*. Regardless, our analysis of the *CD25890* mutant reinforces the idea that a direct link exists between the nutritional potential of the environment and sporulation initiation^20–25^.

The presence of a C-terminal domain (DUF1732) (^36^; Fig. 1B) suggests that CD25890 could function as a transcriptional regulator. It remains to be tested whether CD25890 binds to *spo0A* and *sinRR* regulatory regions. Two putative transcriptional regulators are upregulated in the *CD25890* mutant, CD21430 (XRE-family like protein) and CD24890 (transcriptional regulator of the fructose PTS operon) while one is downregulated, CD03700 (also a XRE-family like protein) (Table S3). Whether increased expression of *CD21430* or *CD24890*, or the diminished expression of *CD03700* is the cause of the increased expression of *sinRR’* and/or *spo0A* in the *CD25890* mutant remains to be tested.

In all, our analysis shows that the genomic signature of sporulation within the human intestinal microbiome can be used to identify new genes important during the sporulation process. Further studies are needed to established the function of the other signature genes. This may lead to the identification of new genetic determinants for spore formation whose products could act as important targets for the design of drugs effective against spore-forming bacteria.

## Materials and methods

### Growth conditions and general methods

Bacterial strains and their relevant properties are listed in Table S5. The *Escherichia coli* strain DH5α (Bethesda Research laboratories) was used for molecular cloning, while the strain HB101 (RP4) was used as the donor in *C. difficile* conjugation experiments^54^. Luria–Bertani medium was routinely used for growth and maintenance of *E. coli.* When appropriate, ampicillin (100 µg/ml) or chloramphenicol (15 µg/ml) was added to the culture medium. The *C. difficile* strains used in this study are isogenic derivatives of the wild-type strain 630 $\Delta$*erm*^54^ and were routinely grown anaerobically (5% H_2_, 15% CO_2_, 80% N_2_) at 37ºC in brain heart infusion (BHI) medium (Difco)^42^. Assays for toxin production were done in tryptone yeast extract (30 g/L tryptone; 20 g/L yeast extract) (TY)^40^. When necessary, cefoxitin (25 µg/ml) and thiamphenicol (15 µg /ml) were added to *C. difficile* cultures. A defined minimal media (CDMM)^55^ with 1% agar was used as uracil-free medium when performing genetic selections. Growth kinetic parameters were calculated by fitting the growth curve using the logistic equation (see Eq. 1) which provides parameters like growth rate (µ) and the maximum population (OD_max_ or Y_m_). For curve fitting we used only the measurements between 5 and 12 h, which corresponds to the part of the growth curve that obeys to the logistic model.

1 $$ Y = \frac{{Y_{m} \times Y_{0} }}{{\left( {Y_{m} - Y_{0} } \right) \times e^{{\left( { - \mu \times x} \right)}} + Y_{0} }} $$

Sporulation was tested in liquid Sporulation Medium (SM; for 1L: Bacto tryptone 90 g, Bacto peptone 5 g, (NH_4_)_2_SO_4_ 1 g, Tris base 1.5 g, pH 7;^42^) and/or on 70:30 agar medium (for 1L: Bacto peptone 63 g, proteose peptone 3.5 g, (NH_4_)_2_SO_4_ 0.7 g, Tris base 1.06 g, brain heart infusion extract 11.1 g, yeast extract 1.5 g, cysteine 0.3 g and agar 15 g;^43^). Sporulation was regularly induced by inoculating in SM liquid. To determine the total number of cells, the cells were serially diluted and plated on BHI with 0.1% taurocholate (Sigma-Aldrich) to ensure efficient spore germination. To determine the number of spores, the cells were heat killed by incubation for 30 min at 70 °C prior to plating on BHI with 0.1% taurocholate.

### Construction of the *CD25890* mutant

A *CD25890* in-frame deletion mutant was generated using allele-couple exchange (ACE) in *C. difficile* 630 $\Delta$*erm* $\Delta$*pyrE* as described^37^. The homology regions upstream and downstream of the desired junction point within *CD25890* were PCR-amplified and cloned into pMTL-YN3 as follows^37^. The upstream fragment (742 bp) was amplified using the primers *CD25890*_AscI_Fwd and *CD25890*_LHA_Rev and the downstream fragment (735 bp) was amplified using the primers *CD25890*_RHA_Fwd and *CD25890*_SbfI_Rev. The fragments were then joined by overlapping PCR, the resulting fragment cleaved with AscI and SbfI and cloned between the same sites of pMTL-YN3, yielding pAM37. This plasmid was introduced into *E. coli* HB101 (RP4) and then transferred to strain 630 $\Delta$*erm* $\Delta$*pyrE* by conjugation^37^. Following two passages on BHI agar supplemented with 25 µg/mL cefoxitin and 15 µg/mL thiamphenicol, colonies that were noticeably larger (indicative of plasmid integration) were screened by colony PCR to identify single-crossover mutants using primers flanking the upstream and downstream homology regions in conjunction with a plasmid-specific primer (P3 with P2 and P4 with P1) to amplify across the integration junction (Fig. S2). Clones positives for single crossover mutants were streaked onto *C. difficile* minimal medium (CDMM) supplemented with 5 µg/mL uracil and 2 mg/mL 5-fluoroorotic acid (FOA) to select for plasmid excision. The isolated FOA-resistant colonies were screened by PCR using primers P3 and P4. Double-crossover mutants, in which the mutant allele was successfully integrated yielded products smaller than those seen in WT revertants (1630 bp instead of 2472 bp). In order to restore the *pyrE*^+^ phenotype, plasmid pMTL-YN1 carrying the WT *pyrE* allele was conjugated into the isolated double-crossover mutants. The resulting colonies were restreaked onto non-supplemented CDMM agar to select for uracil prototrophy indicating successful allele exchange. Successful restoration of the WT *pyrE* allele was confirmed by colony PCR using primers flanking the *pyrE* locus (P5 and P6, Fig. S2).

### ***In trans*** complementation of $\Delta$***CD25890*** mutant

To complement the *CD25890* mutation the coding sequence and its expected promoter region were amplified by PCR using primers *CD25890* _Fwd_BamHI and *CD25890* _Rev_XhoI yielding a 1515 bp fragment. The fragment was then digested with BamHI and XhoI and inserted between the same sites of pMTL-YN1^37^, yielding pAM38. This plasmid was introduced into *E. coli* HB101 (RP4) and then transferred to strain 630 $\Delta$*erm* $\Delta$*pyrE* $\Delta$*CD25890* by conjugation^37^. Following two passages on BHI agar supplemented with 25 µg/mL cefoxitin and 15 µg/mL thiamphenicol, colonies that were noticeably larger (indicative of plasmid integration) were streaked onto onto non-supplemented CDMM to select for uracil prototrophy indicating successful allele exchange. Successful restoration of the WT *pyrE* allele was confirmed by colony PCR using primers flanking the *pyrE* locus (P5 and P6, Fig. S2).

### Construction of P_***tet***_-***sinRR’***

To construct a P_*tet*_-*sinRR’* fusion, the *sinRR’* operon was amplified by PCR using primers IMV503 and IMV505, yielding a 762 bp fragment. The fragment was then digested with BamHI and SacI and inserted between the same sites of pRPF185^56^, yielding pDIA5972.

### Biofilm formation assays

For the biofilm assay, 1 ml of BHI-S medium containing 0.1 M glucose, 0.1% cysteine and DOC (240 $\upmu$M) was inoculated in a well of a 24-well microplate^39^. Microplates were incubated at 37 °C for 24 h. The biofilm was washed with PBS (Phosphate buffered saline), stained with 1 ml of crystal violet (0.2%) followed by two washes with PBS. The OD600nm was measured after resuspension of the cells in methanol/acetone using non-inoculated medium as a negative control.

### ***SNAP***^***Cd***^ transcriptional fusions

To construct transcriptional *SNAP*^*Cd*^ fusions to the *spo0A* promoter, a 568 bp DNA fragment containing the Spo0A promoter region was PCR-amplified using genomic DNA from strain 630∆*erm* and primer pairs CDspo0A_598_Fw/CDspo0A_SNAP_Rev. These fragments were cloned into pFT47^41^ to create pMS463 (Table S7). Plasmid pMS463 was transferred to 630∆*erm*, and congenic ∆*CD25890* mutants by conjugation from derivatives of *E. coli* HB101 (RP4) (Table S5)*.*

### SNAP labelling, fluorescence microscopy and image analysis

Samples of 1 ml were withdrawn from SM cultures at the desired times following inoculation, and the cells collected by centrifugation (10 min, 4000xg, at 4ºC). The cells were washed with 1 ml of phosphate-buffered saline (PBS; 137 mM NaCl, 10 mM Phosphate, 2.7 mM KCl, pH 7.4), and resuspended in 0.1 ml of PBS supplemented with the lipophilic styryl membrane dye *N*-(3-triethylammoniumprpyl)-4-(*p*-diethylaminophenyl-hexatrienyl) pyridinium dibromide (FM4-64, Molecular Probes, Invitrogen; 10 $\upmu$g.ml^−1^)^57^. For SNAP labelling, TMR-Star was added to cells in culture samples inside an anaerobic chamber to a final concentration of 250 nM (New England Biolabs) and the mixture incubated for 30 min in the dark. Following labelling, the cells were collected by centrifugation (4000xg for 5 min), washed four times with 1 ml of PBS, and finally suspended in 10-20 μl of PBS. For phase contrast and fluorescence microscopy, cells were mounted on 1.7% agarose coated glass slides and observed on a Leica DM6000B microscope equipped with a phase contrast Uplan F1 100 × objective and captured with a CCD Andor Ixon camera (Andor Technologies). Images were acquired and analysed using the Metamorph software suite (version 5.8; Molecular Devices; URL: https://www.moleculardevices.com/products/cellular-imaging-systems/acquisition-and-analysis-software/metamorph-microscopy) and adjusted and cropped using Photoshop S4. Statistical analysis was carried out using GraphPad Prism (Version 7.0; GraphPad Software Inc.). The non-parametric Kolmogorov–Smirnov test (KS-test) was applied to compare distributions obtained from quantifications of the SNAP-TMR signal. The P-value is indicated for all comparisons whose differences were found to be statistically significant. Although the results presented are from a single experiment, all experiments involving quantification of a fluorescence signal were performed independently three times and only results that were considered statistically significant by a KS-test in all three experiments were considered to be statistically relevant.

### His_6_-CD25890 overproduction purification and polyclonal antibody production

A DNA fragment encoding the *CD25890* gene was generated by PCR from *C. difficile* 630∆*erm* genomic DNA using primers CD25890_BamHI_Fw/ CD25890_NotI_Rev. The resulting DNA fragment was cut with BamHI and NotI and cloned between the same sites of pETDuet-1 (Novagen) to produce pDM35. Plasmid pDM35 was introduced into BL21 (DE3) cells and the *E. coli* strain was grown in autoinduction medium. The cells were then harvested by centrifugation (4000 × *g*, for 10 min, at 4ºC) and the sediment resuspended in lysis buffer (20 mM phosphate pH7.4, 1 mM PMSF, 10 mM Imidazole). The suspension was lysed using a French pressure cell (at 18,000 lb/in^2^) and the lysate cleared by centrifugation (15,000 × *g*, 30 min at at 4ºC), and the supernantant was loaded onto a 1 ml Histrap column (Amersham Phamarcia Biotech). The bound protein was eluted with a discontinuous imidazole gradient and the fractions containing His_6_-CD25890 were identified by SDS-PAGE. The antibody was produced by Eurogentec (Seraing, Belgium).

### Preparation of C*. difficile* extracts and immunoblotting

Whole cell extracts were obtained by withdrawing 20 ml samples from SM or TY cultures of *C. difficile* 6, 8, 10 and 12 h of after inoculation. The cells were collected by centrifugation (4000xg, for 5 min at 4ºC), the cell sediment was washed with phosphate-buffered saline (PBS) and suspended in 1 ml French press buffer (10 mM Tris pH 8.0, 10 mM MgCl2, 0.5 mM EDTA, 0.2 mM NaCl, 10% Glycerol, 1 mM PMSF). The cells were lysed using a French pressure cell (18,000 lb/in2). Proteins in the extracts were resolved on 12% SDS-PAGE gels. Anti-CD25890 were used at a 1:1000 dilution, and an anti-rabbit secondary antibody conjugated to horseradish peroxidase (Sigma) was used at a 1:10,000 dilution. The monoclonal anti-TcdA primary antibody (Santa Cruz Biotechnology) were used at a 1:1000 dilution, and an anti-mouse secondary antibody conjugated to horseradish peroxidase (Sigma) was used at a 1:2000 dilution. The immunoblots were developed with enhanced chemiluminescence reagents (Amersham Pharmacia Biotech). Images were adjusted and cropped and quantified using ImageJ (http://rsbweb.nih.gov/ij/).

### Detection of Spo0A phosphorylation using a Phos-tag acrylamide gel

Phos-tag acrylamide gels were prepared according to the instructions provided (Wako); 10% acrylamide gels were copolymerized with 25 nM Phos-tag acrylamide and 10 nM MnCl2. 20-ml of bacterial cultures were centrifuged at 5,000 g at 4 °C for 10 min, and the pellets suspended in 1 ml 10 mM Tris pH 8.0. The cells were lysed using a French pressure cell (18,000 lb/in2). Samples were stored on ice prior to loading onto Phos-tag acrylamide gels and run at 4 °C. Gels were fixed for 10 min in transfer buffer with 10 mM EDTA and then washed for 10 min in transfer buffer without EDTA twice. After transfer to a nitrocellulose membrane, the samples were probed with rabbit polyclonal anti-Spo0A or anti-FliC antibodies at a 1:1000 dilution and an anti-rabbit secondary antibody conjugated to horseradish peroxidase (Sigma) was used at dilution 1:10,000. Images were adjusted and cropped and quantified using ImageJ (http://rsbweb.nih.gov/ij/). In order to dephosphorylate Spo0A ~ P, samples were incubated at 100 °C for 5 min.

### RNA extraction and RNA-sequencing

RNA for RNA-Seq was extracted from two independent biological replicates of WT and *CD25890* mutant *C. difficile* strains, after 10 h of growth in SM, using a RNeasy Kit (Qiagen). Contaminating genomic DNA was depleted by two DNase treatments (Promega), according to manufacturer's recommendations. DNAse-treated RNA (5 µg) was mRNA enriched using a Ribo-Zero Magnetic Kit (Epicentre). After Illumina sequencing the reads were mapped to *C. difficile 630* genome using Hisat. Statistical analyses were performed with DESeq2. A gene was considered differentially expressed when the fold change was > 2 and the adjusted p value was < 0.01.

### Animal studies

Animals were euthanized in accordance with the Panel on Euthanasia of the American Veterinary Medical Association guidelines. All animal studies were performed with prior approval from the Emory University Institutional Animal Care and Use Committee (IACUC), and in compliance with the ARRIVE guidelines. Animal Infection studies were performed using the Syrian golden hamster model (*Mesocricetus auratus*) as previously described^58^. Briefly, 6–8 week old male and female hamsters were purchased from Charles River Laboratories and were housed in within an ABSL-2 facility in the Emory University Division of Animal Resources. Hamsters were housed in individual sterile cages and fed sterile water and rodent feed, ad libitum. Animals were administered 30 mg/kg clindamycin by oral gavage seven days before inoculation with approximately 5000 *C. difficile* spores, as indicated. Spores were prepared as previously described and stored in PBS solution with 1% BSA^59,60^. Prior to inoculation, spores were heated at 60 °C for 20 min and allowed to cool to room temperature before administering to animals by oral gavage. Following infection, animals were monitored at least once per day throughout the experiment for signs of disease. Hamsters were considered moribund when they became severely lethargic or had lost 15% or more of their maximum body weight. Fecal samples and animal weights were collected daily; cecal samples were collected post-mortem. Fecal and cecal contents were enumerated by plating on TCCFA medium^42,61^. Differences in survival times were statistically assessed by log-rank regression, while bacterial counts were evaluated using a one-way ANOVA with the Dunnett’s multiple-comparison test. Statistical analyses were performed using GraphPad Prism v.7 for Macintosh. Spores were decontaminated from workspace surfaces as previously described^62^.

### qPCR

After 12 h of growth on 70:30 agar plates, cells from WT, $\Delta$*CD25890*, and *CD25890*^*C*^ were harvested for RNA extraction and subsequent cDNA synthesis as previously described^22,63^. Harvested cells were mixed with a 1:1 ethanol: acetone solution and stored at -80C overnight. A total of 10ug of RNA was extracted and treated with DNAse I for synthesis of 1ug cDNA (PCR machine used). qPCR primers were generated using the IDT PrimerQuest Tool (Integrated DNA Technologies) and can be found in Table S6. qPCR reactions were carried out on a Roche LightCycler 96 instrument and performed in technical triplicate. For relative quantification, the $\Delta \Delta$Ct method was used to normalize *dapF*, *CD25890*, and *gmk* expression to that of the internal control, *rpoC*^64^.

## Supplementary Information

Supplementary Information
