# Supplementary material for: CD25890, a conserved protein that modulates sporulation initiation in Clostridioides difficile
Source: Sci Rep. 2021 Apr 12;11:7887. doi: 10.1038/s41598-021-86878-9 (PMC8041843; doi:10.1038/s41598-021-86878-9)
Supplement: Supplementary file 1 — Supplementary Information [file 41598_2021_86878_MOESM1_ESM.pdf]

Supplementary Information

CD25890, a conserved protein that modulates sporulation initiation in *Clostridioides difficile*

Diogo Martins[1], Michael A. DiCandia[2], Aristides L. Mendes[1], Daniela Wetzel[2], Shonna M. McBride[2], Adriano O. Henriques[1] and Mónica Serrano[1]*

[1]Instituto de Tecnologia Química e Biológica António Xavier, Avenida da República, 2780-157 Oeiras, Portugal

[2]Department of Microbiology and Immunology, Emory University School of Medicine, Atlanta, Georgia, USA

***Corresponding author**: Telf. +351-21-4469524; Fax. +351-21-4411277; e-mail address: serrano@itqb.unl.pt

Running title: Modulation of sporulation initiation in *C. difficile*

Key words: Sporulation; *Clostridioides difficile*; Spo0A; YicC-family protein

Supplementary Information

Figure S5 to S7 correspond to full-length Coomassie-stained gels and full-length blots displayed in Figures 3 to 4.

Supplementary figures legends

Figure S1. Genomic signature of sporulation. A: The inner circle contains the signature defined as those genes present in 90% of sporulating bacteria and in no more than 10% of the remaining bacterial species [1]. The outer circle has the remaining genes identified in a genomic signature of sporulation within the human intestinal microbiome [2]. Both signatures are enriched with known sporulation-associated genes involved in spore morphogenesis and germination. Genes not specifically associated with a sporulation stage or uncharacterized genes are shown inside the circles. The positions of the genes are shown in degrees in the *C. difficile* 630 chromosome. The *CD25890* gene is a signature gene not previously characterized (blue arrow). **B:** Neighbourhood analysis of genes surrounding *CD25890* in the genomes of the indicated sporulating bacteria. Genes are colour coded to indicate conservation in the various genomes. Grey genes are not conserved with other genomes in the *CD25890* neighbourhood.

Figure S2. Construction of the Δ*CD25890* in-frame deletion mutant using allele-coupled exchange (ACE). A: PCR fragments A, B and C were obtained with primers pairs P2/P3, P1/P4 and P3/P4, respectively. PCR fragments D and E were obtained with primers P5/P6. Note that following the initial single reciprocal cross-over, only the integration represented first (of the two possible orientations represented) was obtained. The shorter *CD25890* gene (in yellow) represents the allele with an in-frame deletion of codons 5 to 291 of the 293-codon open reading frame. **B:** PCR analysis of the parental 630Δ*erm* strain in comparison

with the *CD25890* deletion mutant (Δ*CD25890*) and the *CD25890* in trans complementation strain (*CD25890*^{C}, with a copy of the *CD25890* gene at the *pyrE* locus). PCR analysis was performed using the primer pair P3/P4 (for *CD25890* locus verification) and P5/P6 (for *pyrE* locus verification). The position and sizes of the expected products is shown on the right side of the panel (red arrows). The position on molecular size markers (M, in bp) is shown on the left side of the panel. **C:** Quantification of the expression of the indicated genes (*dapF*, *CD25890* and *gmk*) by qRT-PCR in the WT, Δ*CD25890* mutant and the complemented strain. The graph shows the fold change of the differential expression of *dapF*, *CD25890* and *gmk* between the Δ*CD25890* or the complemented strain and the WT.

Figure S3. Disruption of the Δ*CD25890* gene has no effect on growth, biofilm formation or toxin production. A: Mean values of the OD_{600} measured after crystal violet staining of the mass of biofilm obtained after 24 h in the absence or in the presence of DOC (final concentration of 240 μm). One-way ANOVA was performed to determine statistically significant differences. The data represent the mean ± SD of at least three independent experiments performed in triplicate. **B:** The WT and the Δ*CD25890* and Δ*tcdAB* mutants were grown in TY and samples were collected 8, 10, 12 and 14 hours after inoculation. Extracts were prepared and proteins (15 μg) resolved by SDS-PAGE and subjected to immunobloting using an anti-TcdA antibody (upper panel)**.** In the bottom panel the same extracts were loaded in a SDS-PAGE and subjected to Coomassie blue staining, as a loading control. The position of molecular weight markers (in kDa) is indicated on the left side of the panels and the arrow on the right indicates the position of TcdA. The parenthesis indicates possible degradation products. **C:** Samples of an SM liquid culture of the wild type strain (WT, 630 Δ*erm*), the Δ*CD25890* mutant and the complementation strain (*CD25890*^{C}) were collected at 14 hours after inoculation,

stained with the membrane dye FM4-64 and examined by phase contrast and fluorescent microscopy. The numbers in the panels are the percentage of sporulating cells. Data shown are from one representative experiment in which at least 100 cells were analysed for each strain. Scale bar, 1 µm. Images were acquired by Metamorph™ (version 5.8; Molecular Devices) **D:** The optical density of cultures of the WT and the Δ*CD25890* strains was measured at 600nm in intervals of 2 hours until 20 hours hour after inoculation. The data represent the mean ± standard deviation (SD) of three independent experiments.

Figure S4. The Δ*CD25890* phenotype in 70:30 sporulation medium. A: Cells were grown in 70:30 sporulation medium plates and the titer of heat resistant spores and total viable cells measured 12 hours following inoculation. The data in the graph represent the mean and standard deviation of two independent experiments. **B:** Quantitative analysis of the fluorescence intensity (FI.) of *C. difficile* cells carrying a P_{spoOA}-$SNAP^{Cd}$ transcriptional fusion in the WT and congenic Δ*CD25890* mutant. Only cells with no signs of sporulation were quantified. Cells that The cells were collected after 10 h of growth in liquid SM (left) or in plates of 70:30 sporulation medium (right) , stained with TMR-Star, and examined by fluorescence microscopy to monitor SNAP production. Data shown are from one experiment, and are representative of at least three independent experiments. The numbers in the legend represent the mean and the SD of fluorescence intensity. Images were analysed by Metamorph™ (version 5.8; Molecular Devices).

Figure S5. Accumulation of CD25890 during growth. Full-length Coomassie-stained gels (left) and full-length blots (right) for the panels A and B of Figure 3. The wild type strain (WT), the Δ*CD25890* mutant, the complementation strain (*CD25890*^{C}) (**A**) and the *spo0A* mutant (**B**) were grown in SM and samples

were collected at 6, 8, 10 and 12 hours after inoculation for western blot analysis using anti-CD25890 antibody (The parenthesis indicates the position of cross-reactive species).

Figure S6. Increased expression of *spo0A* in the Δ*CD25890* mutant. Full-length Coomassie-stained gels (left) and full-length blots (right) for the panel C of Figure 4. **A and B:** Samples were collected from the wild type strain (WT), the Δ*CD25890* mutant and the complementation strain (*CD25890*^{C}) grown in liquid SM, at the indicated times after inoculation. Extracts were prepared and proteins (15 µg) resolved by SDS-PAGE and subjected to immunobloting using an anti-Spo0A antibody (The parenthesis indicates the position of cross-reactive species, * degradation products).

Figure S7. Phosphorylation of Spo0A in the Δ*CD25890* mutant. Full-length blots for the panels A and C of Figure 5. **A:** Samples were collected from the WT and the Δ*CD25890* mutant grown in liquid SM, at the indicated times after inoculation. Extracts were prepared and proteins (15 µg) resolved by Phos-tag SDS-PAGE and subjected to immunobloting using an anti-Spo0A antibody**.** In panel B the same extracts were loaded in a SDS-PAGE and subjected to immunoblotting using an anti- FliC antibody, as a loading control. **C:** Extracts were prepared from liquid SM cultures of the P_{tet}-*spo0A* and Δ*CD25890* P_{tet}-*spo0A* strains grown in the presence (50 nM) or in the absence of anhydrotetracycline, 8 hours after inoculation. Proteins (15 µg) were resolved by Phos-tag SDS-PAGE and subject to immunoblotting using an anti-Spo0A antibody. In **A** and **C**: The faster migrating bands (black arrows) show the unphosphorylated form of Spo0A (Spo0A), and the slower migrating bands indicate the phosphorylated form of Spo0A (Spo0A~P). The samples heated at 100°C for 5 min were loaded as a control for the position of unphosphorylated Spo0A.

Supplementary Tables

Table S1 – Sporulation efficiency of the Δ*CD25890* mutant. The total viable cell count and the spore titer was determined 12, 24, 48 and 72 hours following inoculation into SM sporulation medium. The heat resistant spore counts were determined by assessing the CFU/ml before and after incubation at 70ºC for 10 min. The results shown are averages and standard deviations for three biological replicates.

	12h		24h		48h		72h	
	Total viable cells	Spore titer	Total viable cells	Spore titer	Total viable cells	Spore titer	Total viable cells	Spore titer
WT	$1.6x10^{8}±7.4x10^{7}$	$7.5x10^{3}±9.8x10^{2}$	$1.7x10^{8}±8.7x10^{7}$	$1.0x10^{4}±1.2x10^{3}$	$8.5x10^{7}±1.1x10^{7}$	$1.3x10^{5}±5.1x10^{4}$	$1.0x10^{7}±1.3x10^{6}$	$1.7x10^{5}±5.8x10^{4}$
Δ*CD25890*	$1.6x10^{8}±2.8x10^{7}$	$3.1x10^{4}±5.3x10^{3}$	$1.3x10^{8}±6.5x10^{7}$	$8.5x10^{4}±6.2x10^{4}$	$3.0x10^{7}±2.1x10^{7}$	$1.5x10^{6}±1.0x10^{5}$	$3.0x10^{7}±9.8x10^{6}$	$2.2x10^{6}±1.2x10^{6}$
$CD25890^{C}$	$1.6x10^{8}±7.1x10^{6}$	$1.8x10^{4}±7.8x10^{3}$	$2.3x10^{8}±1.4x10^{8}$	$1.5x10^{4}±6.8x10^{3}$	$3.0x10^{7}±1.7x10^{7}$	$7.8x10^{4}±2.0x10^{4}$	$1.0x10^{7}±5.3x10^{6}$	$6.0x10^{4}±2.0x10^{4}$

Table S2– Sporulation efficiency of the P*tet-spo0A* alleles in the wild-type and the *ΔCD25890* mutant. The total viable cell count and the spore titer was determined 24 hours following inoculation into SM sporulation medium supplemented with 50 nM of anhydrotetracycline. The heat resistant spore count by determining the cfu/mL obtained after treatment at 70ºC. The results shown are averages and standard deviations for three biological replicates.

	Total viable cells	Spore titer
WT P_{tet}-*spo0A*	$2.1x10^7±1.3x10^6$	$6.8x10^6±1.3x10^5$
ΔCD25890 P_{tet-} *spo0A*	$2.2x10^7±1.6x10^6$	$7.6x10^6±1.7x10^5$

Table S3. Summary of RNA-Seq data analysis.

T10

Adjusted p-value <0.01

**>4 fold**												**>4 fold**	
GeneID	Base mean	log2 (FC)	StdErr	Wald-Stats	p-value	p-adj	Gene ID	Counts-y1	Counts-y2	Counts-wt1	Counts-wt2	GeneID	
CD630_25890	742,2377	8,814003	0,554696	15,88978	7,46E-57	2,88E-53	CD630_25	0,900608	0	1451,091	1516,959068	**CD630_25890**	csiA
CD630_22150	393,896	-5,297177	0,437403	-12,11053	9,29E-34	1,79E-30	CD630_22	835,7638	710,8656	16,957658	11,99704008	**CD630_22150**	sinR2
CD630_23990	1754,292	-4,359196	0,385452	-11,30932	1,18E-29	1,52E-26	CD630_23	4395,865	2354,944	128,39369	137,9659609	**CD630_23990**	conserved hypotetical protein K CotJB1?
CD630_25990	520,8707	-4,2162	0,383798	-10,98546	4,49E-28	4,33E-25	CD630_25	1066,319	928,0566	48,45045	40,65663583	**CD630_25990**	transcriptional regulator **G**-ClnR activates this (Shonna)
CD630_22140	642,0691	-4,512416	0,419085	-10,7673	4,91E-27	3,79E-24	CD630_22	1451,779	1037,619	24,225225	54,6531826	**CD630_22140**	sinR1
CD630_21152	77,26572	-4,714773	0,605147	-7,791119	6,64E-15	7,76E-13	CD630_21	145,8984	159,832	0	3,332511134	**CD630_21152**	hypothetical protein
**>3 fold**												**>3fold**	
GeneID	Base mean	log2 (FC)	StdErr	Wald-Stats	p-value	p-adj	Gene ID	Counts-y1	Counts-y2	Counts-wt1	Counts-wt2	**GeneID**	
CD630_14860	9622,313	-3,362623	0,336005	-10,00765	1,41E-23	9,07E-21	CD630_14	15613,83	19808,85	1717,5685	1349,000507	**CD630_14860**	Putative ribosome recycling factor **G**
CD630_02140	895,2066	-3,925211	0,395009	-9,93701	2,87E-23	1,58E-20	CD630_02	2205,588	1192,295	89,633333	93,31031175	**CD630_02140**	hypothetical protein **F/G and E**
CD630_26871	13714,77	-3,577978	0,377022	-9,490096	2,31E-21	1,11E-18	CD630_26	25996,04	25207,69	2444,3252	1211,034546	**CD630_26871**	hypothetical protein **F/G**
CD630_35800	5113,604	-3,396744	0,362004	-9,383165	6,40E-21	2,74E-18	CD630_35	10667,7	8221,679	973,85405	591,1874752	**CD630_35800**	hypothetical protein **K and spoIIID**-spore coat?
CD630_05430	1178,604	-3,22409	0,354386	-9,097682	9,23E-20	3,56E-17	CD630_05	2298,35	2006,278	239,82973	169,9580678	**CD630_05430**	hypothetical protein **G**
CD630_12900	594,8953	-3,609153	0,400431	-9,013167	2,00E-19	6,43E-17	CD630_12	1426,562	801,0933	79,943243	71,98224049	**CD630_12900**	SAPS - **G**
CD630_21501	1424,791	-3,872208	0,429461	-9,01644	1,94E-19	6,43E-17	CD630_21	1843,544	3559,484	203,49189	92,64380952	**CD630_21501**	hypothetical protein
CD630_21410	2683,488	-3,025213	0,337194	-8,971742	2,92E-19	8,66E-17	CD630_21	5490,103	4176,899	518,41982	548,5313326	**CD630_21410**	serine-type D-Ala-D-Ala carboxypeptidase **F**
CD630_23150	594,4266	-3,463466	0,391056	-8,8567	8,24E-19	2,27E-16	CD630_23	1169,889	1034,396	111,43604	61,98470709	**CD630_23150**	hypothetical protein **G**
CD630_21120	11299,03	-3,029037	0,342325	-8,848421	8,88E-19	2,28E-16	CD630_21	16350,53	24376,31	2151,2	2318,094745	**CD630_21120**	hypothetical protein **G**
CD630_28090	19464,41	-3,417222	0,39121	-8,735001	2,44E-18	5,22E-16	CD630_28	45950,8	26191,82	3662,854	2052,160356	**CD630_28090**	hypothetical protein **G**
CD630_24430	654,3255	-3,828389	0,443969	-8,623105	6,52E-18	1,32E-15	CD630_24	689,8654	1791,02	75,098198	61,31820486	**CD630_24430**	hypothetical protein **E**
CD630_10631	3448,476	-3,02777	0,36924	-8,200015	2,40E-16	4,03E-14	CD630_10	6224,999	6222,49	874,53063	471,8835766	**CD630_10631**	hypothetical protein **K**
CD630_17260	7795,376	-3,174629	0,390852	-8,122323	4,57E-16	7,06E-14	CD630_17	15049,15	13425,89	1865,3423	841,1258102	**CD630_17260**	hypothetical protein **E**
CD630_10280	993,3143	-3,430317	0,43196	-7,941276	2,00E-15	2,66E-13	CD630_10	1849,848	1840,001	210,75946	72,64874272	**CD630_10280**	diguanylate cyclase **G** GGDEF domain
CD630_02130	1078,832	-3,044146	0,385342	-7,899848	2,79E-15	3,47E-13	CD630_02	2383,908	1517,759	254,36486	159,2940322	**CD630_02130**	cotF **E**
CD630_32510	6993,698	-3,129061	0,409523	-7,640736	2,16E-14	2,31E-12	CD630_32	12039,32	13470,35	1773,2865	691,8293114	**CD630_32510**	dehydrogenase **E**
CD630_17880	688,6987	-3,078522	0,414255	-7,431474	1,07E-13	1,04E-11	CD630_17	1331,098	1168,449	179,26667	75,98125385	**CD630_17880**	hypothetical protein **G**
CD630_23950	486,486	-3,155362	0,443962	-7,107285	1,18E-12	8,61E-11	CD630_23	533,1597	1247,72	101,74595	63,31771154	**CD630_23950**	hypothetical protein **E**
CD630_31510	166,071	-3,304611	0,470207	-7,027989	2,10E-12	1,39E-10	CD630_31	223,3507	391,2016	29,07027	20,66156903	**CD630_31510**	hypothetical protein **E**
CD630_20550	249,3705	-3,423286	0,492975	-6,944135	3,81E-12	2,37E-10	CD630_20	434,0928	493,6745	55,718018	13,99654676	**CD630_20550**	hypothetical protein **E/K**
CD630_11930	4175,981	-3,04417	0,444086	-6,854905	7,14E-12	4,33E-10	CD630_11	5698,144	9493,89	1131,318	380,5727715	**CD630_11930**	SpoIIIAB **E**
CD630_26860	798,0271	-3,109758	0,456333	-6,814674	9,45E-12	5,36E-10	CD630_26	1371,625	1542,894	215,6045	61,98470709	**CD630_26860**	membrane protein **F**
CD630_19400	9059,59	-3,077314	0,465039	-6,61733	3,66E-11	1,88E-09	CD630_19	12253,67	20856,78	2446,7477	681,1652758	**CD630_19400**	membrane protein (DUF3866 superfamily) **E**
CD630_31501	103,7792	-3,194542	0,490113	-6,517975	7,13E-11	3,51E-09	CD630_31	173,8173	210,1017	14,535135	16,66255567	**CD630_31501**	hypothetical protein **E**
CD630_21430	153,2186	-3,048391	0,471204	-6,469364	9,84E-11	4,69E-09	CD630_21	246,7665	309,3522	38,76036	17,99556012	**CD630_21430**	HTH-type transcriptional regulator
CD630_19060	26,87575	-3,028447	0,693738	-4,365401	1,27E-05	0,000149	CD630_19	31,52126	70,89321	2,4225225	2,666008907	**CD630_19060**	hypothetical protein
**>2 fold**												**>2 fold**	
GeneID	Base mean	log2 (FC)	StdErr	Wald-Stats	p-value	p-adj	Gene ID	Counts-y1	Counts-y2	Counts-wt1	Counts-wt2		
CD630_12300	20680,33	-2,869729	0,324574	-8,841513	9,44E-19	2,28E-16	CD630_12	39712,29	33837,97	4244,2594	4926,78446	**CD630_12300**	sigK **E**
CD630_34640	29339,44	-2,803163	0,320076	-8,757802	1,99E-18	4,52E-16	CD630_34	50247,6	53492,15	7267,5675	6350,433217	**CD630_34640**	GerS **E**
CD630_29670	2620,63	-2,763155	0,334397	-8,263098	1,42E-16	2,74E-14	CD630_29	5184,798	4060,247	605,63063	631,844111	**CD630_29670**	spoVFB **K**
CD630_13540	791,8847	-2,976955	0,361686	-8,230778	1,86E-16	3,42E-14	CD630_13	1598,578	1251,587	138,08378	179,289099	**CD630_13540**	hypothetical protein **G**
CD630_11330	9609,206	-2,787066	0,339158	-8,217596	2,08E-16	3,64E-14	CD630_11	17669,02	16304,79	2638,127	1824,883097	**CD630_11330**	hypothetical protein **K**
CD630_32490	167579,3	-2,906712	0,355665	-8,172623	3,02E-16	4,85E-14	CD630_32	343540,3	255558,4	27725,77	43492,60281	**CD630_32490**	sspB **G**
CD630_14300	9383,753	-2,906269	0,359867	-8,075951	6,70E-16	9,93E-14	CD630_14	21308,37	12249,06	1930,7504	2046,828338	**CD630_14300**	pdaA **G**
CD630_16310	2197,314	-2,620308	0,326755	-8,019174	1,06E-15	1,52E-13	CD630_16	3932,052	3715,449	576,56036	565,1938883	**CD630_16310**	sodA **G**
CD630_29680	2104,228	-2,630319	0,330091	-7,968475	1,61E-15	2,21E-13	CD630_29	3916,742	3415,119	557,18018	527,8697636	**CD630_29680**	dpaA **K**
CD630_19270	934,7499	-2,856239	0,361344	-7,904477	2,69E-15	3,46E-13	CD630_19	1964,225	1364,372	220,44955	189,9531346	**CD630_19270**	ytlC ABC-transporter **K**
CD630_14330	17127,92	-2,745794	0,349562	-7,854963	4,00E-15	4,82E-13	CD630_14	37205,9	23194,32	3897,8387	4213,627078	**CD630_14330**	cotE **K**
CD630_30240	1413,497	-2,870177	0,372563	-7,703869	1,32E-14	1,50E-12	CD630_30	3207,063	1838,068	314,92793	293,927482	**CD630_30240**	hypothetical protein **Spo0A**
CD630_10650	18787,27	-2,451948	0,318604	-7,695898	1,41E-14	1,55E-12	CD630_10	32804,63	31459,18	5179,3531	5705,925563	**CD630_10650**	hypothetical protein **K**
CD630_21420	814,0315	-2,637333	0,349973	-7,535809	4,85E-14	5,06E-12	CD630_21	1490,505	1350,193	230,13964	185,287619	**CD630_21420**	transporter
CD630_08960	1426,276	-2,643703	0,354057	-7,466886	8,21E-14	8,33E-12	CD630_08	2952,191	2032,057	387,6036	333,2511134	**CD630_08960**	hypothetical protein **K**
CD630_00160	5194,459	-2,662615	0,360283	-7,390342	1,46E-13	1,32E-11	CD630_00	6671,701	11527,24	1264,5568	1314,342391	**CD630_00160**	dnaX **E**
CD630_23750	42988,53	-2,98851	0,404197	-7,393702	1,43E-13	1,32E-11	CD630_23	87879,48	67369,17	11787,995	4917,453429	**CD630_23750**	hypothetical protein **F/G**

CD630_32980	10860,1	-2,53465	0,342983	-7,390024	1,47E-13	1,32E-11	CD630_32	19111,79	18417,41	3561,1081	2350,086852	**CD630_32980**	ATP/GTP-binding protein **E**
CD630_10660	3212,055	-2,499543	0,338588	-7,382247	1,56E-13	1,36E-11	CD630_10	4642,632	6415,836	886,64324	903,1105173	**CD630_10660**	hypothetical protein **E**
CD630_14020	1267,386	-2,823889	0,382827	-7,376414	1,63E-13	1,39E-11	CD630_14	2071,397	2434,215	370,64594	193,2856458	**CD630_14020**	glpQ **E**
CD630_35670	86019,72	-2,371246	0,322222	-7,359041	1,85E-13	1,55E-11	CD630_35	149099,2	142670	28660,864	23648,83201	**CD630_35670**	SipL **E**
CD630_24351	1217,934	-2,71313	0,371159	-7,309885	2,67E-13	2,19E-11	CD630_24	2360,492	1925,073	368,22342	217,9462282	**CD630_24351**	hypothetical protein (ykz-like) **Spo0A**
CD630_33500	4170,258	-2,874745	0,394439	-7,288179	3,14E-13	2,52E-11	CD630_33	10066,99	4855,54	726,75675	1031,745447	**CD630_33500**	family 2 glycosyl transferase **E/K**
CD630_28410	3111,832	-2,368394	0,328958	-7,199682	6,04E-13	4,75E-11	CD630_28	5798,111	4759,512	939,93874	949,7656732	**CD630_28410**	amidohydrolase **G**
CD630_03110	78187,15	-2,369678	0,33128	-7,153098	8,48E-13	6,54E-11	CD630_03	146914,3	118437,4	21015,383	26381,49114	**CD630_03110**	putative coat assembly protein **E**
CD630_28160	3572,195	-2,872964	0,402637	-7,135377	9,65E-13	7,30E-11	CD630_28	6624,869	6156,109	1065,9099	441,8909764	**CD630_28160**	hypothetical protein **E**
CD630_07930	334,9444	-2,938587	0,413129	-7,113	1,14E-12	8,42E-11	CD630_07	660,1453	542,0108	89,633333	47,98816033	**CD630_07930**	putative membrane protein **G**
CD630_24310	4233,107	-2,592759	0,36593	-7,085399	1,39E-12	9,72E-11	CD630_24	9465,385	5275,744	1128,8955	1062,404549	**CD630_24310**	nitrite/sulfite reductase **G**
CD630_19040	932,2796	-2,47184	0,34922	-7,078166	1,46E-12	1,01E-10	CD630_19	1418,457	1783,286	283,43513	243,939815	**CD630_19040**	ABC transporter permease **K**
CD630_24000	2836,408	-2,448441	0,347678	-7,042275	1,89E-12	1,28E-10	CD630_24	5760,286	3959,064	881,7982	744,4829873	**CD630_24000**	spore coat peptide assembly protein CotJB2 **K**
CD630_12340	3118,927	-2,341405	0,333753	-7,015386	2,29E-12	1,50E-10	CD630_12	4619,216	5935,695	915,71351	1005,085358	**CD630_12340**	Recombination directionality factor (skin excision) **E**
CD630_25980	1416,307	-2,473318	0,353977	-6,987227	2,80E-12	1,80E-10	CD630_25	2838,715	2028,19	445,74414	352,579678	**CD630_25980**	oligosaccharide deacetylase **G**
CD630_30320	1481,673	-2,355464	0,338696	-6,954504	3,54E-12	2,24E-10	CD630_30	2790,983	2231,847	469,96937	433,8929496	**CD630_30320**	pyridoxal phosphate-dependent transferase **K**
CD630_15110	43496,67	-2,287961	0,333819	-6,853903	7,19E-12	4,33E-10	CD630_15	75482,62	70804,92	16134	11565,14664	**CD630_15110**	cotB **E**
CD630_09020	1636,358	-2,587071	0,37786	-6,846637	7,56E-12	4,49E-10	CD630_09	3169,238	2527,021	549,91261	299,2594998	**CD630_09020**	cation efflux protein **K**
CD630_18840	5428,798	-2,297262	0,335849	-6,840155	7,91E-12	4,62E-10	CD630_18	8987,162	9297,967	1436,5559	1993,50816	**CD630_18840**	hypothetical protein **E**
CD630_11680	7795,605	-2,469875	0,36129	-6,836272	8,13E-12	4,68E-10	CD630_11	13137,16	13665,63	2798,0135	1581,609784	**CD630_11680**	YlbJ-like protein/spore cortex formation **E**
CD630_05710	5744,36	-2,211921	0,327411	-6,755795	1,42E-11	7,94E-10	CD630_05	9884,168	9249,63	2129,3973	1714,243727	**CD630_05710**	hypothetical protein **Spo0A**
CD630_32480	8660,042	-2,290529	0,341755	-6,702257	2,05E-11	1,13E-09	CD630_32	14167,46	14982,31	3284,9405	2205,455868	**CD630_32480**	polysaccharide deacetylase **E**
CD630_15430	4088,598	-2,281584	0,342183	-6,667739	2,60E-11	1,41E-09	CD630_15	8091,058	5661,145	1218,5288	1383,658623	**CD630_15430**	FMN-dependent NADH-azoreductase **G**
CD630_34650	7949,406	-2,129524	0,319504	-6,665099	2,64E-11	1,42E-09	CD630_34	12594,1	13600,54	2860,9991	2741,990161	**CD630_34650**	hypothetical protein **E**
CD630_34990	27666,14	-2,117466	0,317808	-6,662719	2,69E-11	1,42E-09	CD630_34	47650,24	43363,44	9777,3009	9873,563987	**CD630_34990**	SpoVT **G**
CD630_26570	877,2733	-2,31436	0,348633	-6,638378	3,17E-11	1,65E-09	CD630_26	1315,788	1648,589	256,78739	287,928962	**CD630_26570**	hypothetical protein SpoVD operon **Spo0A**
CD630_21840	1806,296	-2,187269	0,331922	-6,589702	4,41E-11	2,24E-09	CD630_21	2809,895	3190,194	649,23603	575,8579239	**CD630_21840**	Putative N-acetylmuramoyl-L-alanine amidase **K**
CD630_10670	52616,34	-2,094633	0,318793	-6,570514	5,01E-11	2,51E-09	CD630_10	91483,71	81110,86	19421,363	18449,44814	**CD630_10670**	CdeC **K**
CD630_21590	480,8561	-2,769941	0,425058	-6,516613	7,19E-11	3,51E-09	CD630_21	734,8957	970,5925	152,61892	65,31721822	**CD630_21590**	putative membrane protein
CD630_34570	4990,15	-2,110668	0,324168	-6,511024	7,46E-11	3,60E-09	CD630_34	8598,1	7810,498	1894,4126	1657,591038	**CD630_34570**	hypothetical protein **E**
CD630_01060	6406,289	-2,124442	0,331179	-6,414779	1,41E-10	6,63E-09	CD630_01	10860,43	10253,09	2565,4513	1946,186502	**CD630_01060**	cwlD **E**
CD630_10310	1687,181	-2,311032	0,361489	-6,393092	1,63E-10	7,46E-09	CD630_10	2754,958	2943,357	656,5036	393,902816	**CD630_10310**	cell wall anchored protein **F**
CD630_21440	593,6612	-2,56057	0,401525	-6,37711	1,80E-10	8,19E-09	CD630_21	988,8671	1073,71	210,75946	101,3083385	**CD630_21440**	membrane protein **E/K**
CD630_10633	3616,416	-2,19056	0,344507	-6,358538	2,04E-10	9,13E-09	CD630_10	6250,216	5779,086	1455,936	980,4247756	**CD630_10633**	hypothetical protein **E/K** (operon)
CD630_01960	3211,861	-2,070255	0,325802	-6,354327	2,09E-10	9,28E-09	CD630_01	4873,187	5635,366	1145,8531	1193,038986	**CD630_01960**	pseudogene **K**
CD630_19280	3801,19	-2,91274	0,461114	-6,316749	2,67E-10	1,17E-08	CD630_19	5328,895	8394,401	1155,5432	325,9195889	**CD630_19280**	membrane protein **E**
CD630_10450	1540,333	-2,181796	0,348792	-6,255293	3,97E-10	1,72E-08	CD630_10	3027,842	2093,928	525,68739	513,8732168	**CD630_10450**	sporulation integral membrane protein **Spo0A**
CD630_30230	979,3503	-2,44551	0,39392	-6,208131	5,36E-10	2,30E-08	CD630_30	2223,6	1148,47	237,40721	307,9240288	**CD630_30230**	hypothetical protein
CD630_28450	6532,16	-2,341367	0,377961	-6,194737	5,84E-10	2,45E-08	CD630_28	14642,08	7550,771	1756,3288	2179,462282	**CD630_28450**	Rbr rubrerythrin **G**
CD630_03140	3295,233	-2,56584	0,417418	-6,146931	7,90E-10	3,24E-08	CD630_03	5027,191	6469,328	1218,5288	465,8850565	**CD630_03140**	membrane protein **E**
CD630_24010	7651,238	-2,132895	0,347596	-6,136131	8,46E-10	3,43E-08	CD630_24	15297,72	9987,564	2463,7054	2855,962042	**CD630_24010**	CotD manganese catalase **K**
CD630_33120	1103,208	-2,246171	0,366456	-6,129435	8,82E-10	3,54E-08	CD630_33	2179,47	1520,337	419,0964	293,927482	**CD630_33120**	major facilitator superfamily transporter **G**
CD630_21021	337,5277	-2,893533	0,473368	-6,112652	9,80E-10	3,90E-08	CD630_21	575,4882	638,0389	106,59099	29,9926002	**CD630_21021**	hypothetical protein
CD630_26360	2631,326	-2,489513	0,408507	-6,094178	1,10E-09	4,33E-08	CD630_26	5998,947	3114,145	491,77207	920,4395752	**CD630_26360**	membrane protein F/**G**
CD630_34620	14282,78	-2,009038	0,329969	-6,08856	1,14E-09	4,44E-08	CD630_34	24927,92	21436,17	4731,1865	6035,844166	**CD630_34620**	mazE antitoxin endoAI **E**
CD630_11950	6191,056	-2,286807	0,376664	-6,07121	1,27E-09	4,85E-08	CD630_11	8540,461	12354,11	2480,6631	1388,990641	**CD630_11950**	SpoIIIAD E
CD630_24890	1116,023	-2,267613	0,373513	-6,071047	1,27E-09	4,85E-08	CD630_24	2247,016	1507,447	426,36396	283,2634464	**CD630_24890**	PTS operon transcription antiterminator
CD630_19981	291,6189	-2,740359	0,451571	-6,068498	1,29E-09	4,88E-08	CD630_19	450,3038	582,6133	96,900901	36,65762247	**CD630_19981**	pseudogene
CD630_24740	883,3688	-2,343502	0,388464	-6,032735	1,61E-09	5,98E-08	CD630_24	1850,748	1150,403	327,04054	205,2826858	**CD630_24740**	DNA polymerase III subunit delta
CD630_14630	13773,21	-2,129003	0,35411	-6,012266	1,83E-09	6,72E-08	CD630_14	21974,82	23537,83	5995,7432	3584,448976	**CD630_14630**	hypothetical protein **G**
CD630_10630	3394,009	-2,42459	0,404156	-5,999139	1,98E-09	7,22E-08	CD630_10	4324,717	7334,869	1283,9369	632,5106132	**CD630_10630**	hypothetical protein **E/K** (operon)
CD630_05720	7400,121	-2,160309	0,3602	-5,997519	2,00E-09	7,22E-08	CD630_05	11068,47	13492,91	3166,2369	1872,871257	**CD630_05720**	YtxC Sporulation protein
CD630_15600	2551,136	-2,500288	0,418397	-5,975871	2,29E-09	8,12E-08	CD630_15	3627,647	5215,807	973,85405	387,2377938	**CD630_15600**	hypothetical protein
CD630_06950	3564,209	-2,205552	0,370463	-5,953501	2,62E-09	9,12E-08	CD630_06	5776,497	6127,751	1533,4568	819,1312367	**CD630_06950**	metalloendopeptidase
CD630_34920	5836,338	-2,532283	0,426426	-5,938391	2,88E-09	9,91E-08	CD630_34	9581,563	10739,03	2252,9459	771,8095786	**CD630_34920**	DivIC septum formation initiation protein
CD630_07600	4372,152	-2,129077	0,358748	-5,93474	2,94E-09	1,00E-07	CD630_07	7150,824	7299,424	1918,6378	1119,723741	**CD630_07600**	Ca2+/Na+ antiporter **E**
CD630_06750	321,0522	-2,528265	0,431598	-5,857913	4,69E-09	1,56E-07	CD630_06	498,036	615,482	118,7036	51,98717369	**CD630_06750**	acetyltransferase
CD630_26350	3737,509	-2,111593	0,361229	-5,845585	5,05E-09	1,66E-07	CD630_26	7247,189	5089,488	1034,4171	1578,943775	**CD630_26350**	Putative membrane protein **G**
CD630_34160	192,2695	-2,402965	0,413603	-5,809829	6,25E-09	2,01E-07	CD630_34	310,7096	350,5991	48,45045	59,31869818	**CD630_34160**	sugar family ABC transporter permease

CD630_15180	1588,504	-2,007914	0,346406	-5,796426	6,77E-09	2,16E-07	CD630_15	2446,05	2720,366	491,77207	695,8283248	**CD630_15180**	Ferrous iron transport protein FeoA **K**
CD630_34860	883,5554	-2,002262	0,345655	-5,792658	6,93E-09	2,19E-07	CD630_34	1496,81	1370,172	370,64594	296,5934909	**CD630_34860**	membrane protein
CD630_36520	15804,15	-2,25835	0,389932	-5,791648	6,97E-09	2,19E-07	CD630_36	21337,19	31903,23	6644,9793	3331,178129	**CD630_36520**	Putative peptidase M1 family **E**
CD630_16780	696,3766	-2,029921	0,355789	-5,705415	1,16E-08	3,52E-07	CD630_16	1263,552	1010,55	230,13964	281,2639397	**CD630_16780**	membrane protein **Spo0A**
CD630_19050	1522,95	-2,107289	0,370041	-5,694741	1,24E-08	3,69E-07	CD630_19	2071,397	2951,091	654,08108	415,2308873	**CD630_19050**	Site-specific recombinase (putative SpoIVCA homolog) **Spo0A?**
CD630_28000	3760,846	-2,104723	0,370824	-5,675792	1,38E-08	4,06E-07	CD630_28	6626,67	5777,797	1715,1459	923,7720863	**CD630_28000**	Putative membrane protein **E**
CD630_12910	8596,751	-2,13138	0,377259	-5,649651	1,61E-08	4,59E-07	CD630_12	17842,84	10643,65	2274,7486	3625,772114	**CD630_12910**	dacF **G**
CD630_28560	2047,518	-2,301469	0,408679	-5,631482	1,79E-08	4,92E-07	CD630_28	3034,147	3906,216	881,7982	367,9092292	**CD630_28560**	membrane protein **F**
CD630_21210	1103,023	-2,153435	0,383103	-5,621038	1,90E-08	5,19E-07	CD630_21	2297,45	1366,306	445,74414	302,592011	**CD630_21210**	hypothetical protein **E**
CD630_20141	379,6465	-2,370396	0,422808	-5,606314	2,07E-08	5,57E-07	CD630_20	696,1696	598,7254	155,04144	68,64972936	**CD630_20141**	hypothetical protein
CD630_23160	204,8794	-2,276539	0,406973	-5,593835	2,22E-08	5,83E-07	CD630_23	373,7521	320,3084	58,14054	67,3167249	**CD630_23160**	OmpR family **E**
CD630_13151	196,3057	-2,646103	0,473659	-5,586518	2,32E-08	6,04E-07	CD630_13	380,957	309,3522	70,253153	24,66058239	**CD630_13151**	hypothetical protein
CD630_23740	7531,116	-2,40867	0,431515	-5,581889	2,38E-08	6,16E-07	CD630_23	11761,93	14149,64	3163,8144	1049,074505	**CD630_23740**	hypothetical protein **E**
CD630_32970	4995,424	-2,121845	0,380391	-5,578066	2,43E-08	6,25E-07	CD630_32	11072,07	5464,578	1652,1604	1792,89099	**CD630_32970**	hypothetical protein
CD630_11940	823,2558	-2,144331	0,387142	-5,538877	3,04E-08	7,48E-07	CD630_11	949,2403	1785,864	271,32252	286,5959575	**CD630_11940**	SpoIIIAC **E**
CD630_28181	87,69277	-2,848382	0,518344	-5,495158	3,90E-08	9,41E-07	CD630_28	174,7179	140,4975	24,225225	11,33053786	**CD630_28181**	hypothetical protein
CD630_10680	2888,899	-2,353647	0,430447	-5,467916	4,55E-08	1,08E-06	CD630_10	4117,578	5758,462	1240,3315	439,2249674	**CD630_10680**	polysaccharide biosynthesis/spoVB **E**
CD630_28680	1808,09	-2,200094	0,404825	-5,43468	5,49E-08	1,28E-06	CD630_28	4208,539	1849,024	658,92612	515,8727235	**CD630_28680**	oxidoreductase **G**
CD630_19410	7926,392	-2,047055	0,378965	-5,401706	6,60E-08	1,52E-06	CD630_19	17047,6	8929,322	3275,2504	2453,394697	**CD630_19410**	hypothetical protein **Spo0A**
CD630_34391	4070,939	-2,664584	0,494461	-5,38887	7,09E-08	1,59E-06	CD630_34	6685,21	7780,852	1523,7667	293,927482	**CD630_34391**	hypothetical protein
CD630_36361	173,0053	-2,550241	0,473296	-5,388254	7,11E-08	1,59E-06	CD630_36	244,0646	358,9774	62,985585	25,99358684	**CD630_36361**	hypothetical protein **E**
CD630_11740	982,2937	-2,001488	0,379064	-5,280085	1,29E-07	2,72E-06	CD630_11	1549,946	1644,722	477,23694	257,2698595	**CD630_11740**	hypothetical protein
CD630_12420	401,6451	-2,164686	0,411514	-5,260294	1,44E-07	2,98E-06	CD630_12	759,2121	577,4574	179,26667	90,64430284	**CD630_12420**	hypothetical protein
CD630_19510	860,2831	-2,088772	0,401005	-5,208848	1,90E-07	3,83E-06	CD630_19	1520,225	1316,036	414,25135	190,6196369	**CD630_19510**	acyl-CoA N-acyltransferase **K**
CD630_16840	702,296	-2,04725	0,393577	-5,201656	1,98E-07	3,93E-06	CD630_16	947,4391	1353,416	327,04054	181,2886057	**CD630_16840**	radical SAM superfamily protein
CD630_20470	190,0058	-2,396769	0,462286	-5,184601	2,16E-07	4,28E-06	CD630_20	244,0646	407,9582	72,675675	35,32461802	**CD630_20470**	OmpR family,
CD630_22640	394,4814	-2,083905	0,402093	-5,182647	2,19E-07	4,30E-06	CD630_22	638,5307	659,3069	184,11171	95,97632066	**CD630_22640**	hypothetical protein
CD630_24680	1694,924	-2,145206	0,420954	-5,096061	3,47E-07	6,43E-06	CD630_24	2468,565	3177,305	821,23513	312,5895444	**CD630_24680**	hypothetical protein **F**
CD630_11960	9204,655	-2,281769	0,451057	-5,058717	4,22E-07	7,61E-06	CD630_11	10773,07	20535,19	4120,7108	1389,657143	**CD630_11960**	SpoIIIAE **E**
CD630_26600	10539,53	-2,164738	0,431374	-5,018241	5,21E-07	9,27E-06	CD630_26	26401,31	8875,185	3030,5757	3851,049866	**CD630_26600**	M16 family peptidase **F**
CD630_33670	81,81567	-2,493338	0,497122	-5,015549	5,29E-07	9,31E-06	CD630_33	149,5008	136,6306	21,802703	19,32856458	**CD630_33670**	hypothetical protein
CD630_00511	752,3756	-2,05614	0,410131	-5,013377	5,35E-07	9,38E-06	CD630_00	856,4778	1617,01	336,73063	199,2841658	**CD630_00511**	hypothetical protein
CD630_21500	145,7368	-2,207258	0,444601	-4,964576	6,89E-07	1,16E-05	CD630_21	198,1337	291,9511	50,872973	41,98964029	**CD630_21500**	endonuclease/exonuclease/phosphatase
CD630_24780	1160,398	-2,039404	0,419388	-4,862807	1,16E-06	1,88E-05	CD630_24	2503,689	1308,302	552,33513	277,2649263	**CD630_24780**	DNA uptake transporter
CD630_14230	157,9891	-2,01645	0,419794	-4,803428	1,56E-06	2,44E-05	CD630_14	250,3689	267,4607	58,14054	55,98618705	**CD630_14230**	hypothetical protein **Spo0A**
CD630_21860	377,7067	-2,564003	0,533698	-4,80422	1,55E-06	2,44E-05	CD630_21	399,8697	933,8569	147,77387	29,32609798	**CD630_21860**	hypothetical protein
CD630_34540	367,6653	-2,019504	0,44543	-4,533831	5,79E-06	7,60E-05	CD630_34	602,5064	601,9478	196,22432	69,98273381	**CD630_34540**	nucleotide-binding protein
CD630_25630	884,6557	-2,18775	0,484507	-4,515415	6,32E-06	8,26E-05	CD630_25	2283,04	703,7762	385,18108	166,6255567	**CD630_25630**	hypothetical protein
CD630_28390	268,3914	-2,004234	0,453314	-4,421291	9,81E-06	0,000121	CD630_28	392,6649	484,6518	142,92883	53,32017814	**CD630_28390**	two-component sensor histidine kinase
CD630_26650	160,7633	-2,04083	0,462374	-4,41382	1,02E-05	0,000124	CD630_26	214,3446	313,8636	77,520721	37,3241247	**CD630_26650**	AraC family transcriptional regulator
CD630_17060	56,56029	-2,512736	0,571183	-4,399175	1,09E-05	0,000131	CD630_17	82,85589	116,0071	19,38018	7,998026721	**CD630_17060**	thiamine-phosphate pyrophosphorylase ThiE2
CD630_25970	73,80919	-2,23814	0,511135	-4,378773	1,19E-05	0,000142	CD630_25	147,6996	104,4064	21,802703	21,32807126	**CD630_25970**	lspA lipoprotein signal peptidase
CD630_19260	65,14142	-2,384046	0,561565	-4,245364	2,18E-05	0,000241	CD630_19	100,868	124,3854	26,647748	8,664528948	**CD630_19260**	pseudogene **E**
CD630_26870	1482,986	-2,059974	0,491466	-4,191489	2,77E-05	0,000298	CD630_26	2486,577	2445,816	830,92522	168,6250634	**CD630_26870**	hypothetical protein **F**
CD630_02921	60,9862	-2,344477	0,563145	-4,163186	3,14E-05	0,000328	CD630_02	123,3832	87,0053	24,225225	9,331031175	**CD630_02921**	hypothetical protein
CD630_33690	186,2889	-2,118652	0,510064	-4,153699	3,27E-05	0,000339	CD630_33	236,8598	385,4013	96,900901	25,99358684	**CD630_33690**	hypothetical protein
CD630_07380	156,1926	-2,08857	0,510141	-4,094104	4,24E-05	0,000421	CD630_07	183,7239	335,776	79,943243	25,32708462	**CD630_07380**	hypothetical protein
CD630_18210	55,73994	-2,246545	0,562055	-3,997023	6,41E-05	0,00059	CD630_18	78,35285	112,1402	21,802703	10,66403563	**CD630_18210**	transposase-like protein B
EBG00001175819	42,12402	-2,37458	0,603913	-3,931986	8,42E-05	0,000744	EBG00001	87,35893	59,93699	14,535135	6,665022268	**EBG00001175819**	
CD630_01631	46,06692	-2,225907	0,588832	-3,780206	0,000157	0,001259	CD630_01	63,04253	97,96153	7,2675675	15,99605344	**CD630_01631**	hypothetical protein
CD630_15640	45,22533	-2,035705	0,573016	-3,552615	0,000381	0,002612	CD630_15	62,14192	90,22772	14,535135	13,99654676	**CD630_15640**	hypothetical protein
CD630_17452	32,17147	-2,263394	0,638756	-3,543443	0,000395	0,002672	CD630_17	55,83767	56,07008	12,112613	4,665515587	**CD630_17452**	hypothetical protein
EBG00001175749	43,31341	-2,166945	0,617509	-3,509173	0,00045	0,002946	EBG00001	53,13584	94,73911	19,38018	5,998520041	**EBG00001175749**	
EBG00001175906	51,95933	-2,075558	0,616338	-3,367564	0,000758	0,004556	EBG00001	58,53949	116,6516	26,647748	5,998520041	**EBG00001175906**	
CD630_23030	19,2831	-2,34322	0,709018	-3,304879	0,00095	0,005472	CD630_23	28,81944	41,89144	2,4225225	3,999013361	**CD630_23030**	pseudogene
CD630_12341	28,37011	-2,111583	0,678424	-3,112483	0,001855	0,009541	CD630_12	29,72005	68,31528	12,112613	3,332511134	**CD630_12341**	hypothetical protein (skin)

GeneID	Base mean	log2 (FC)	StdErr	Wald-Stats	p-value	p-adj	Gene ID	Counts-y1	Counts-y2	Counts-wt1	Counts-wt2	GeneID	
CD630_20541	3388,689	2,4491154	0,328855	7,447397	9,52E-14	9,42E-12	CD630_205	985,2646	973,1704	5167,241	6429,08	CD630_205	hypothetical protein
EBG0000117568	961,005	2,7381324	0,386394	7,086382	1,38E-12	9,72E-11	EBG000011	284,592	162,4099	2010,694	1386,325	EBG00001175680	
CD630_33790	495,9061	2,3011964	0,359443	6,402122	1,53E-10	7,12E-09	CD630_337	143,1966	165,6323	922,9811	751,8145	CD630_337	conjugative transposon protein
CD630_33830	950,2159	2,1386087	0,34484	6,201741	5,58E-10	2,37E-08	CD630_338	364,746	294,5291	1700,611	1440,978	CD630_338	ATPase
CD630_06052	5175,903	2,0030469	0,335211	5,975476	2,29E-09	8,12E-08	CD630_060	2224,501	1684,68	9098,995	7695,435	CD630_060	hypothetical protein
EBG0000117567	719268,1	2,0929505	0,352718	5,933782	2,96E-09	1,00E-07	EBG000011	274987,9	236343,8	1468729	897011,4	EBG00001175675	
CD630_00370	820,193	2,0215244	0,345894	5,844355	5,09E-09	1,66E-07	CD630_003	267,4804	342,2209	1368,725	1302,345	CD630_003	acetoin dehydrogenase E1 component subunit beta
CD630_33911	1006,281	2,0876129	0,369923	5,643375	1,67E-08	4,73E-07	CD630_339	368,3485	342,8653	2088,214	1225,698	CD630_339	hypothetical protein
CD630_33800	329,6459	2,1228161	0,378394	5,610072	2,02E-08	5,49E-07	CD630_338	120,6814	106,3398	625,0108	466,5516	CD630_338	cell wall hydrolase
CD630_28720	185,5775	2,2579327	0,405231	5,57196	2,52E-08	6,43E-07	CD630_287	53,13584	62,51492	336,7306	289,9285	CD630_287	uxaA-D-galactate dehydratase/altronate hydrolase
CD630_11031	562,9563	2,1024641	0,380489	5,525683	3,28E-08	7,96E-07	CD630_110	169,3142	222,3469	1141,008	719,1559	CD630_110	Transposon protein
CD630_01721	78572,74	2,0393905	0,37383	5,455399	4,89E-08	1,16E-06	CD630_017	37355,4	19806,27	144699,7	112429,6	CD630_017	ferredoxin
CD630_33810	774,1556	2,0296647	0,373064	5,440521	5,31E-08	1,25E-06	CD630_338	261,1762	304,1963	1579,485	951,7652	CD630_338	pseudogene
CD630_31001	297,999	2,1744729	0,403365	5,390838	7,01E-08	1,59E-06	CD630_310	111,6753	84,42737	615,3207	380,5728	CD630_310	hypothetical protein
CD630_26000	5442,931	2,2008025	0,416131	5,28872	1,23E-07	2,62E-06	CD630_260	2106,521	1402,397	5278,677	12984,13	CD630_260	cstA Carbon starvation **IIID**
CD630_14150	246,4029	2,7815404	0,537831	5,171777	2,32E-07	4,52E-06	CD630_141	40,52734	54,13663	750,982	139,9655	CD630_141	membrane protein
CD630_33350	146,735	2,1940253	0,458296	4,787356	1,69E-06	2,61E-05	CD630_333	33,32248	58,00354	307,6604	187,9536	CD630_333	conjugative transposon protein
CD630_28700	103,0117	2,1143094	0,45109	4,687115	2,77E-06	4,03E-05	CD630_287	32,42187	36,09109	169,5766	173,9571	CD630_287	2-keto-3-deoxygluconate permease
CD630_33780	52,93474	2,3099151	0,566148	4,080057	4,50E-05	0,00044	CD630_337	9,006075	18,04554	118,7036	65,98372	CD630_337	conjugative transposon protein
CD630_03700	58,67486	2,0357824	0,523691	3,887375	0,000101	0,000857	CD630_037	22,51519	16,75658	84,78829	110,6394	CD630_037	HTH-type transcriptional regulator
CD630_20451	55,0322	2,2873115	0,646369	3,538706	0,000402	0,002697	CD630_204	5,403645	19,33451	164,7315	30,6591	CD630_204	hypothetical protein

Table S3. Summary of RNA-Seq data analysis.

Table S4– Sporulation efficiency of the P*tet-sinRR'* alleles in wild type and *ΔCD25890* mutant. The total viable cell count and the spore titer were determined 24 hours following inoculation into sporulation medium supplemented with 50 nM of anhydrotetracycline. The heat resistant spore count by determining the cfu/mL obtained after treatment at 70ºC. The results shown are averages and standard deviations for three biological replicates.

	Total viable cells	Spore titer
WT vector	$1.2x10^8 \pm 5.8x10^6$	$5.1x10^5 \pm 2.0x10^5$
ΔCD25890 vector	$1.2x10^8 \pm 2.5x10^7$	$1.8x10^6 \pm 2.5x10^5$
WT P_{tet}-*sinRR'*	$1.0x10^8 \pm 2.0x10^7$	$1.7x10^6 \pm 2.1x10^5$
ΔCD25890 P_{tet-} *sinRR'*	$1.2x10^8 \pm 1.0x10^7$	$2.2x10^6 \pm 1.5x10^5$

Table S5. Bacterial strains used in this study.

Strain	Genotype and phenotype	Origin/Construction
E. coli		
BL21	*ompT gal dcm lon* $hsdS_B(r_B^- m_B^-)$ *λ(DE3 [lacI lacUV5-T7p07 ind1 sam7 nin5]) [malB⁺]* $_{K\text{-}12}(\lambda^S)$	Laboratory Stock
DH5α	*fhuA2 lac(del)U169 phoA glnV44 Φ80' lacZ(del)M15 gyrA96 recA1 relA1 endA1 thi-1 hsdR17*	Laboratory Stock
HB101 (RP4)	*supE*44 *aa*14 *galK*2 *lacY*1 Δ (*gpt-proA*) 62 *rpsL*20 (Str^R)*xyl-5 mtl-1 recA*13 Δ (*mcrC-mrr*) *hsdS*$_B(r_B^- m_B)$ RP4 (Tra^+ IncP Ap^R Km^R Tc^R)	Laboratory Stock
C. difficile		
AHCD 536	630Δ*erm* Δ*spo0A*	[3]
AHCD591	630Δ*erm tcdAB*::*ermB*	[4]
AHCD607	630Δ*erm* P_{spo0A}-$SNAP^{Cd}$	This work
AHCD772	*630ΔermΔpyrE*	[5]
AHCD1014	*630ΔermΔpyrEΔCD25890*	This work
AHCD1055	*630Δerm ΔCD25890 pyrE:: CD25890*	"
AHCD1077	630Δ*erm* Δ*CD25890*	"
AHCD1142	630Δ*erm* Δ*CD25890* P_{spo0A}-$SNAP^{Cd}$	"
AHCD1190	*630Δerm*	"
AHCD1263	630Δ*erm* Δ*CD25890* P_{tet}-*gusA*	"
AHCD1272	630Δ*erm* P_{tet}-*gusA*	"
AHCD1274	630Δ*erm* Δ*CD25890 spo0A::*P_{tet}-*spo0A*	"
AHCD1279	630Δ*erm spo0A::* P_{tet}-*spo0A*	"
AHCD1484	630Δ*erm* P_{tet}-*sinRR'*	"
AHCD1590	630Δ*erm* Δ*CD25890* P_{tet}-*sinRR'*	"

Table S6. Oligonucleotide primers used in this study.

Name	Sequence (5' to 3')
CD25890_AscI_Fwd	ACAGGCGCGCCATAGAAGATTTTCTGTAGGAG
CD25890_LHA_Rev	ATTCTTACTCTATACTTATAGCCATTAATTAAACCTCC
CD25890_RHA_Fwd	ATAGAGTAAGAATACAATATTAGAAGGGG
CD25890_SbfI_Rev	CTTCCTGCAGGCTCTTTGAAACAGTCCCTAC
CD25890_Fwd_BamHI	GAAGGATCCGTAGATACATTAGATGG
CD25890_Rev_XhoI	CAACTCGAGCTTTTCCTGCACCTGATGG
P1	TTCTTTCTATTCAGCACTGTTATGC
P2	CATCAAGAAGAGCGACTTCG
P3	GCAATAGATGGAAGATTCGACC
P4	GCTACACATACAGGCTTTTCC
P5	CAATAATTTTATAACATTAACATGG
P6	ATTTACATTTTTTAAGTAACAC
CD25890_Fw	CGGAATTCGATATAAAAATAAATCTTACC
CD25890_Rev	CACAATCTTTATCCATTAATTAAACCTCCAAAATACC
SNAP_SOE_Fw	ATGGATAAAGATTGTGAAATGAAGAGAACC
XhoI_SNAP_Rev	CCGCTCGAGTTACCCAAGTCCTGGTTTCCCCAAACG
CDspo0A_598D	GCTAGGATCCTTAATGGGTAATTTC
CDspo0A_SNAP_Rev	CCCCATTAAAAACTCGAGTCTTATTACAGA
CD25890 970D	GGAATTCATATGTGAAGGTATTTTGGAGG
CD25890 1920R	ATAAGAATGCGGCCGCCACAAACATTTAAGCATTCCCC
*CD25890*_BamHI_Fw	CGGGATCCGATGGCTATAAGTATGACTGG
*CD25890*_NotI_Rev	GCGGCCGCTTACTCTATATTTTGTATTTGTTC
IMV503	CCCGAGCTCTAATCTAAAGTGGAGGGATA
IMV505	CCGGATCCTACTCATTAGCACTATAAGACAAT

Underlined sequences represent introduced restriction sites.

Table S7. Plasmids used in this study.

Plasmid	Relevant genotype	Origin
pFT47	pMTL84121-*SNAP*^{Cd} (Cm^R/Tm^R)	[6]
pMTL84121	*Clostridia* modular plasmid;*catP* (Cm^R/Tm^R)	[7]
pMTL-YN3	Plasmid for mutant construction through ACE methodology (Cm^R/Tm^R)	[5]
pMTL-YN1	Plasmid for Δ*pyrE* reversion through ACE methodology (Cm^R/Tm^R)	[5]
pMTL-YN1C	Plasmid for *in trans* complementation of mutants (Cm^R/Tm^R)	[5]
pMLD138	pMTL-YN3-P_{tet}-spo0A	[8]
pRPF185	Plamid with the tetracycline-inducible promoter P_{tet}	[9]
pAM25	pMTL84121 - P_{tet}	This work
pAM37	pMTL-YN3 containing homology regions for Δ*CD25890* construction (Cm^R/Tm^R)	"
pAM38	pMTL-YN1C containing *CD25890* (Cm^R/Tm^R)	"
pDIA5972	pRPF185 containing sinRR' (Cm^R/Tm^R)	
pDM35	pETDuet-1-*CD25890* (Amp^R)	"
pMS463	pFT47- P_{spo0A}-*SNAP*^{Cd} (Cm^R/Tm^R)	"

Supplementary References

1 Abecasis, A. B. *et al.* A genomic signature and the identification of new sporulation genes. *J Bacteriol* **195**, 2101-2115, doi:JB.02110-12 [pii]
10.1128/JB.02110-12 (2013).

2 Browne, H. P. *et al.* Culturing of 'unculturable' human microbiota reveals novel taxa and extensive sporulation. *Nature* **533**, 543-546, doi:10.1038/nature17645 (2016).

3 Saujet, L., Monot, M., Dupuy, B., Soutourina, O. & Martin-Verstraete, I. The key sigma factor of transition phase, SigH, controls sporulation, metabolism, and virulence factor expression in Clostridium difficile. *J Bacteriol* **193**, 3186-3196, doi:JB.00272-11 [pii]
10.1128/JB.00272-11 (2011).

4 Kuehne, S. A. *et al.* The role of toxin A and toxin B in Clostridium difficile infection. *Nature* **467**, 711-713, doi:nature09397 [pii]
10.1038/nature09397 (2010).

5 Ng, Y. K. *et al.* Expanding the repertoire of gene tools for precise manipulation of the Clostridium difficile genome: allelic exchange using pyrE alleles. *PLoS One* **8**, e56051, doi:10.1371/journal.pone.0056051
PONE-D-12-24523 [pii] (2013).

6 Pereira, F. C. *et al.* The spore differentiation pathway in the enteric pathogen Clostridium difficile. *PLoS Genet* (2013).

7 Heap, J. T., Pennington, O. J., Cartman, S. T. & Minton, N. P. A modular system for Clostridium shuttle plasmids. *J Microbiol Methods* **78**, 79-85, doi:10.1016/j.mimet.2009.05.004 (2009).

8 Dembek, M. *et al.* Inducible Expression of spo0A as a Universal Tool for Studying Sporulation in Clostridium difficile. *Front Microbiol* **8**, 1793, doi:10.3389/fmicb.2017.01793 (2017).

9 Fagan, R. P. & Fairweather, N. F. Clostridium difficile has two parallel and essential Sec secretion systems. *J Biol Chem* **286**, 27483-27493, doi:10.1074/jbc.M111.263889 (2011).

Martins *et al.* – Figure S1

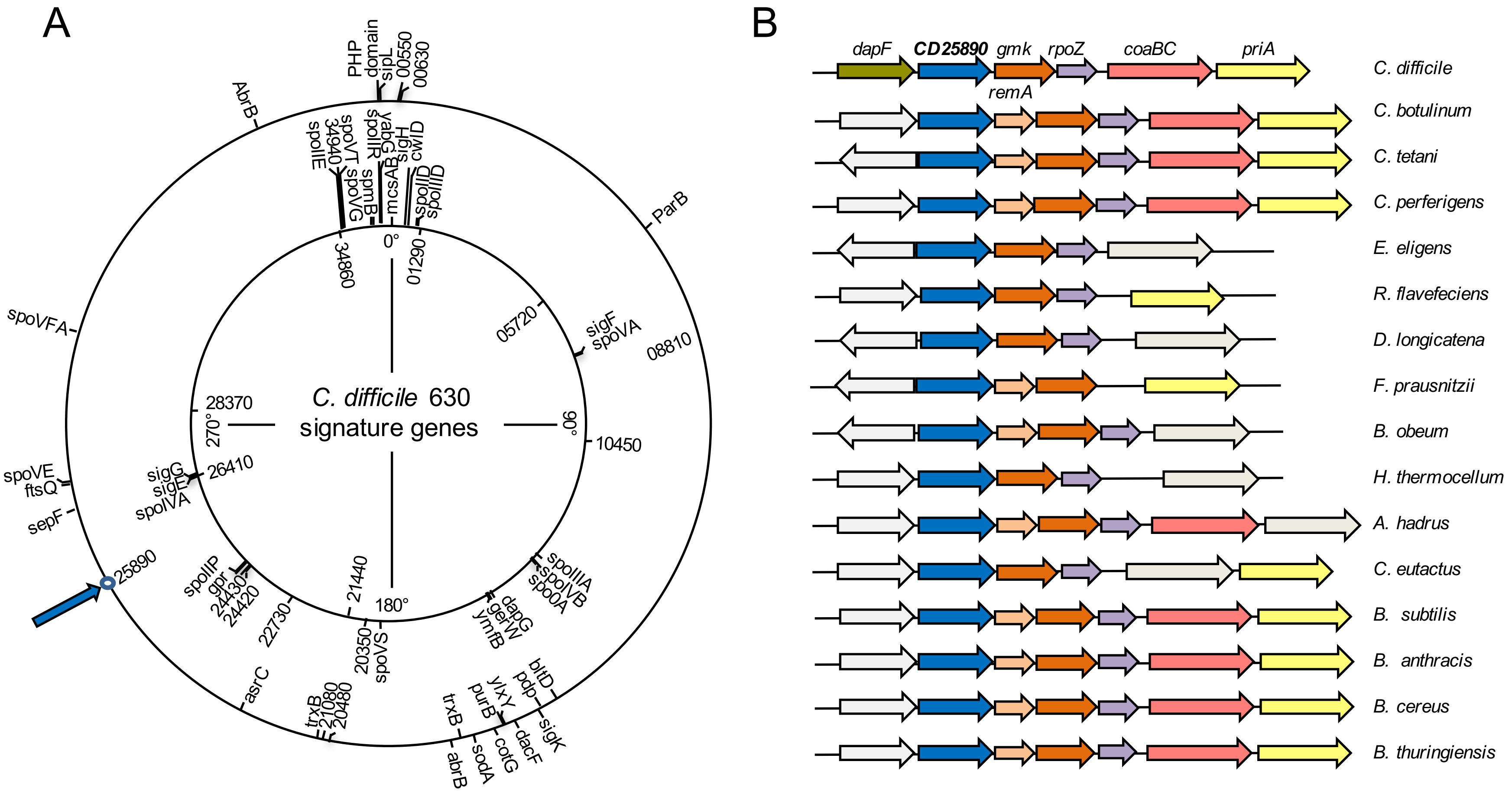

Martins *et al.* – Figure S2

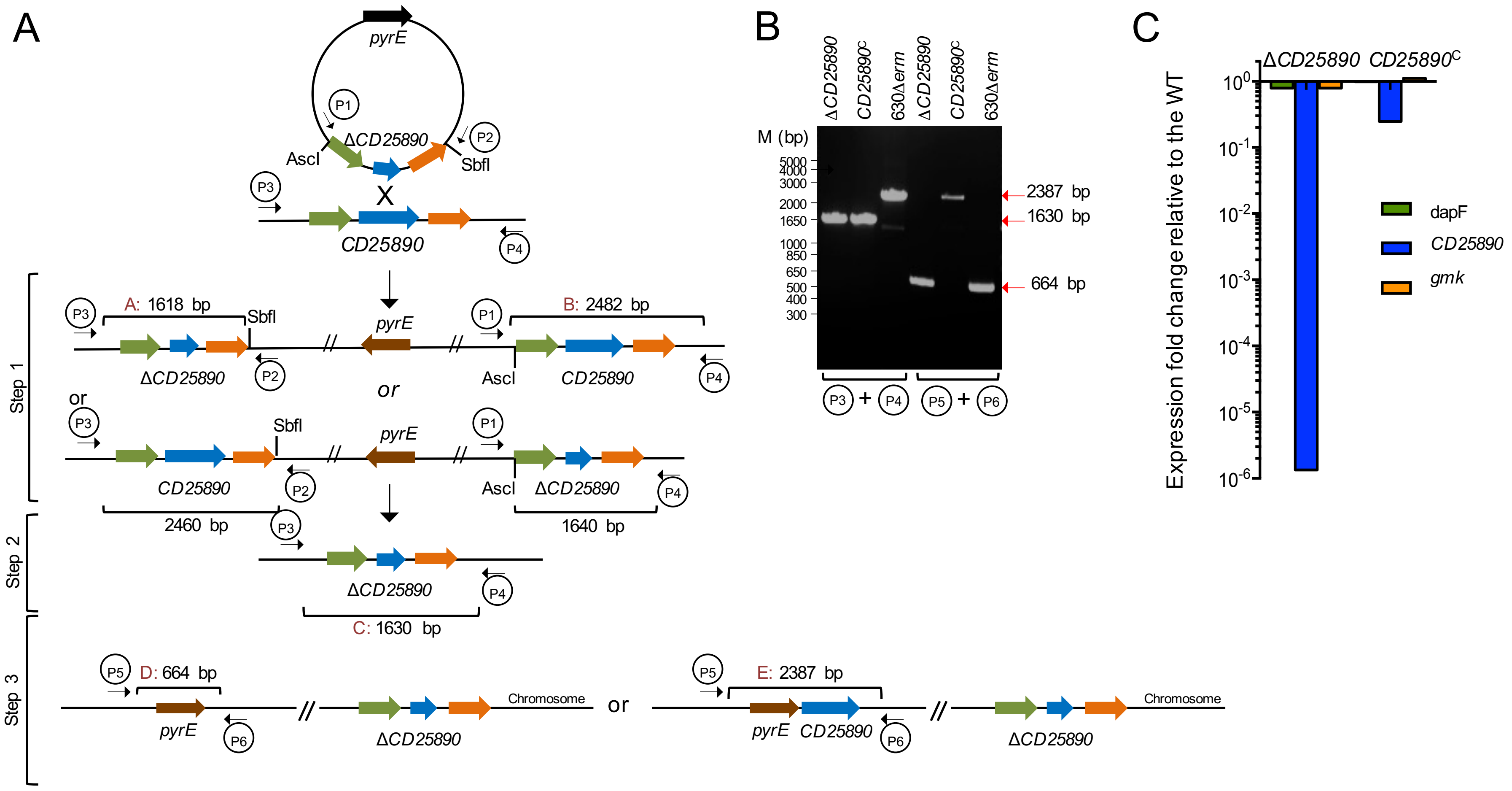

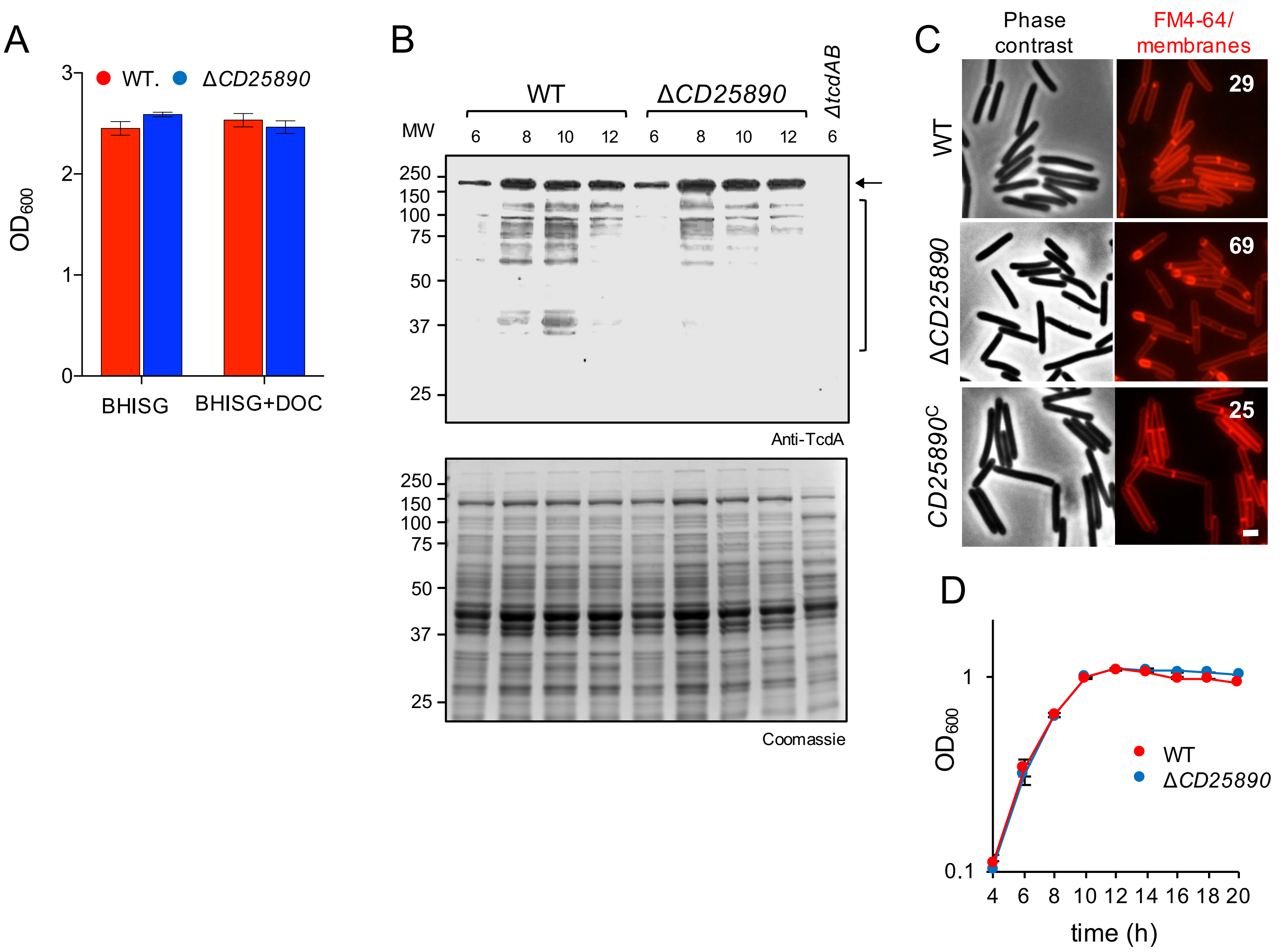
A
OD600
WT.
ΔCD25890
BHISG
BHISG+DOC
B
WT
ΔCD25890
ΔtcdAB
MW
6 8 10 12 6 8 10 12 6
250
150
100
75
50
37
25
Anti-TcdA
Coomassie
C
Phase contrast
FM4-64/ membranes
WT
ΔCD25890
CD25890C
29
69
25
D
OD600
WT
ΔCD25890
time (h)
4 6 8 10 12 14 16 18 20
1
0.1

Martins *et al.* – Figure S4

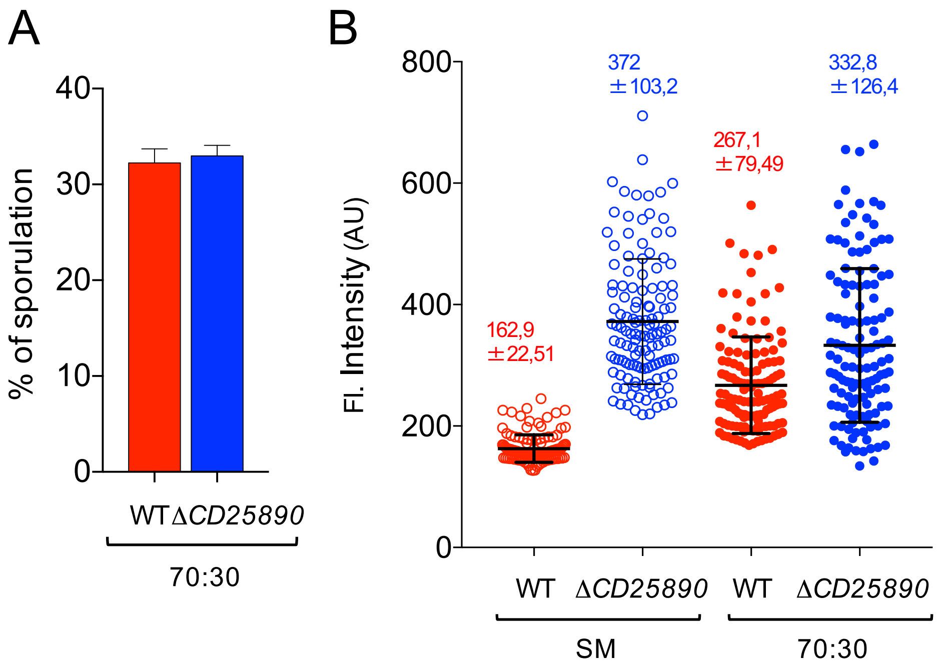

Martins *et al.* – Figure S5

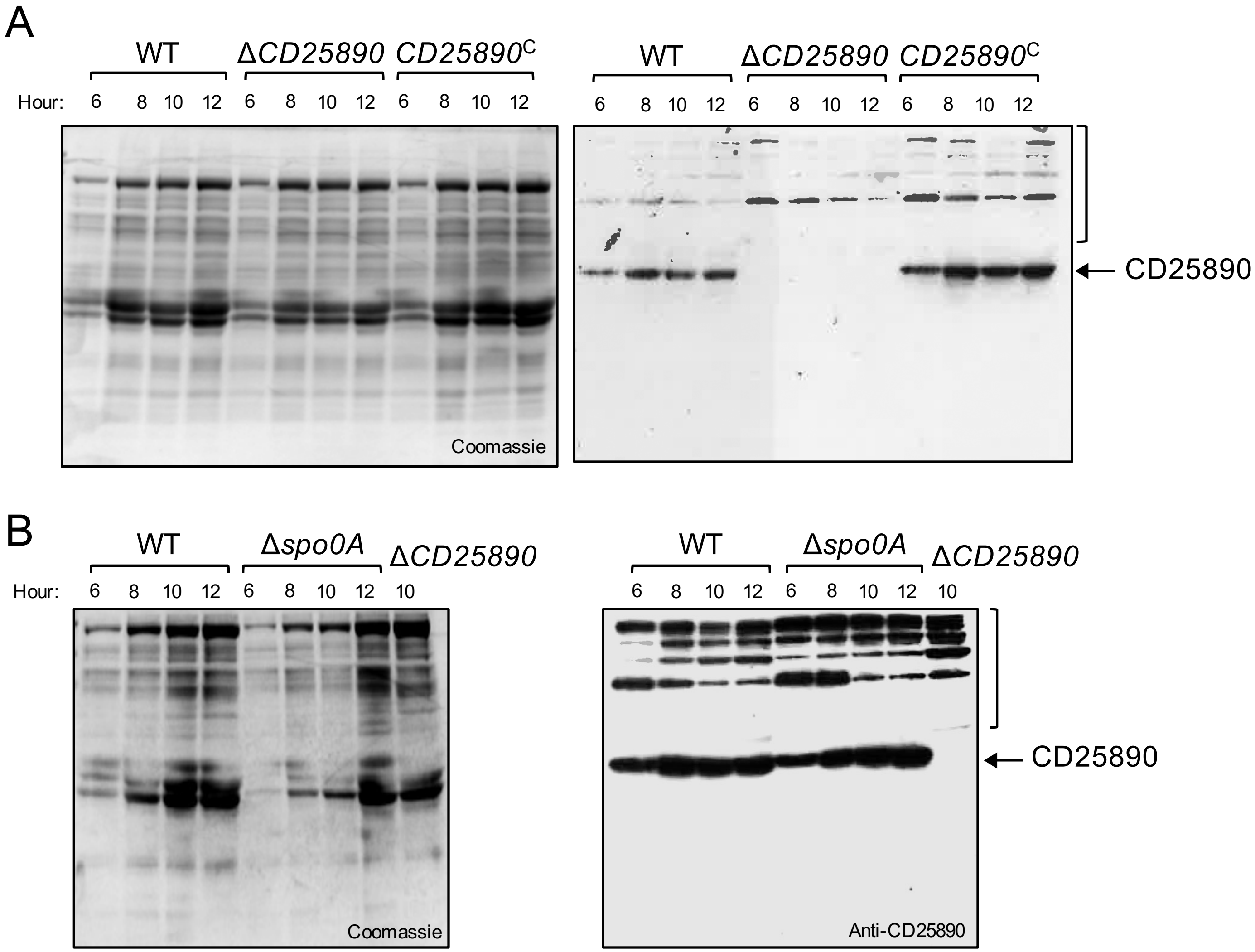

Martins *et al.* – Figure S6

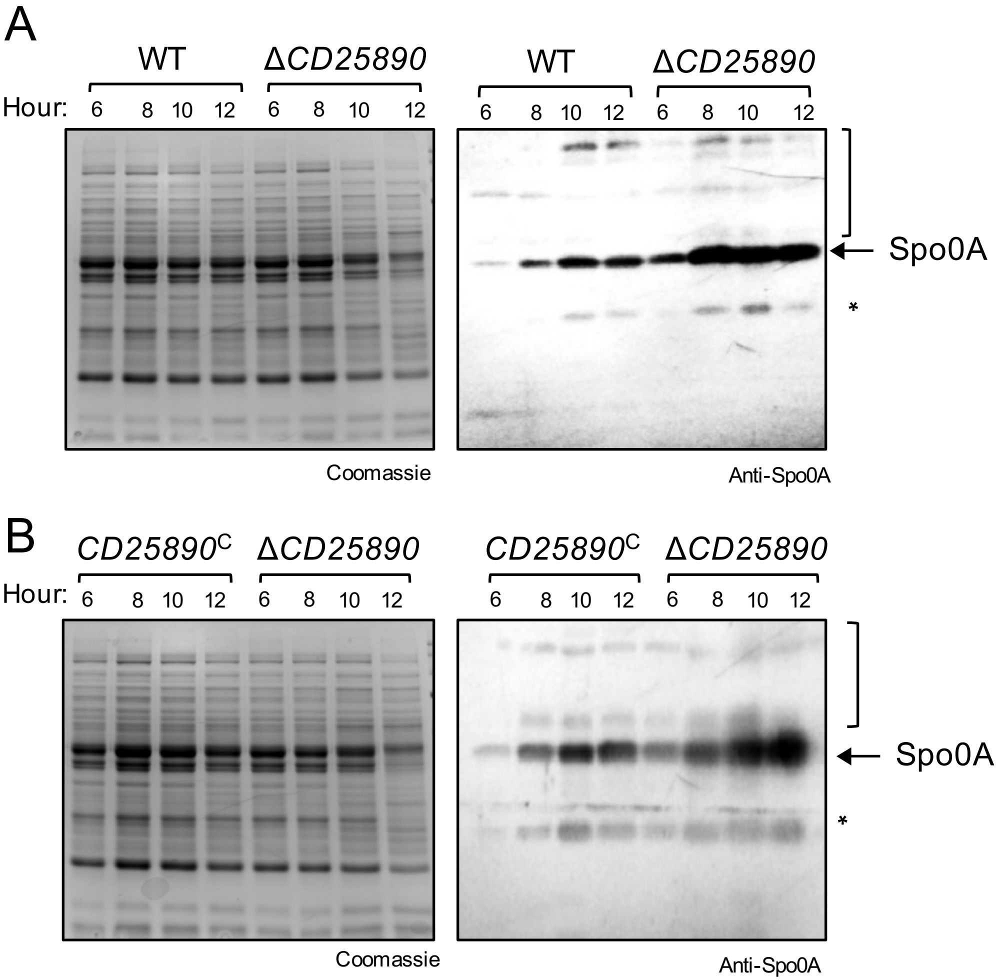

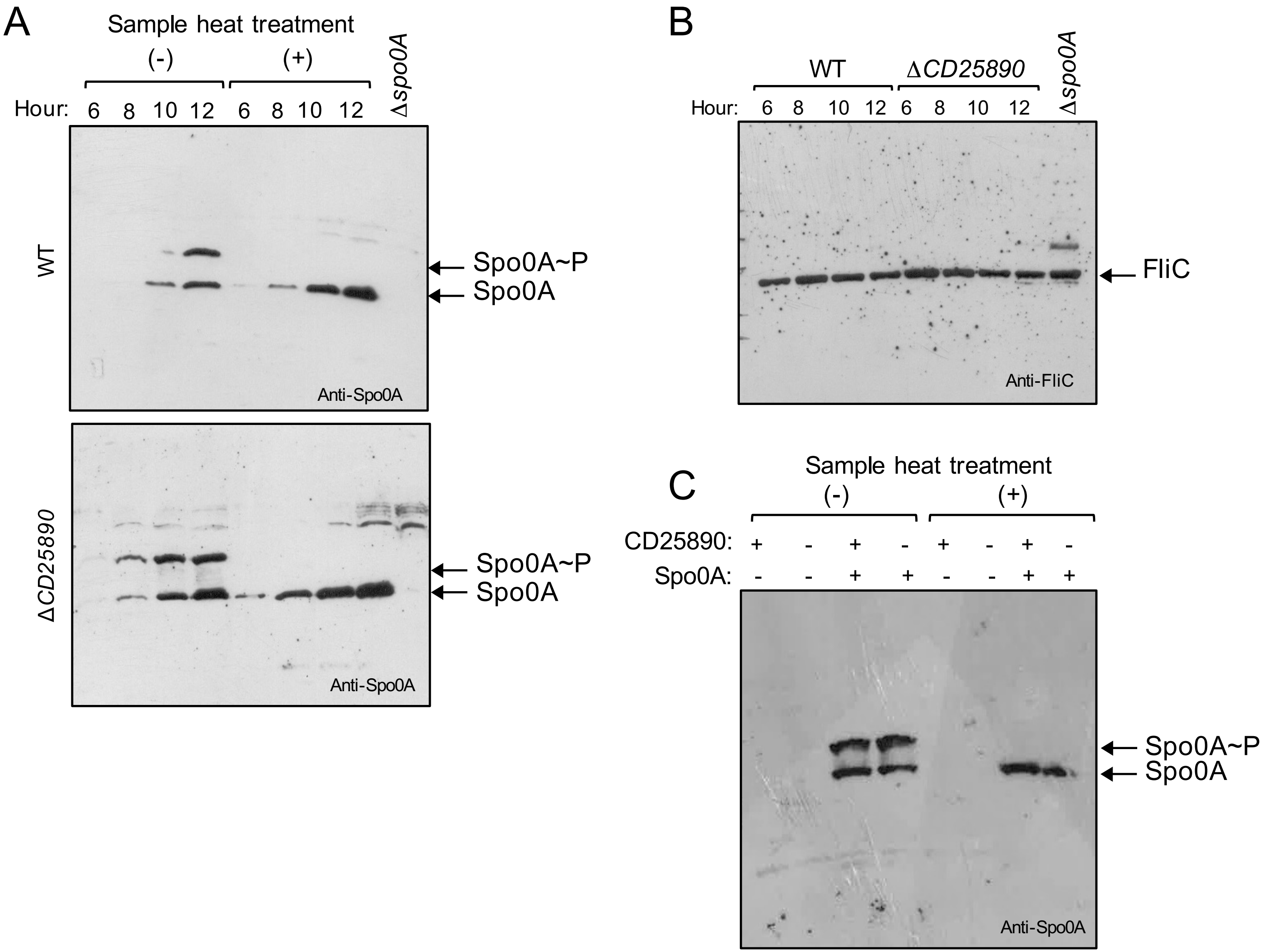
A
Sample heat treatment
(-)
(+)
Δspo0A
Hour: 6 8 10 12 6 8 10 12
WT
ΔCD25890
Spo0A~P
Spo0A
Anti-Spo0A
B
WT
ΔCD25890
Δspo0A
Hour: 6 8 10 12 6 8 10 12
FliC
Anti-FliC
C
Sample heat treatment
(-)
(+)
CD25890: + - + - + - + -
Spo0A: - - + + - - + +
Spo0A~P
Spo0A
Anti-Spo0A
